# Nitric oxide as a source for bacterial triazole biosynthesis

**DOI:** 10.1038/s41467-020-15420-8

**Published:** 2020-03-31

**Authors:** Guiyun Zhao, Yuan-Yang Guo, Shunyu Yao, Xinjie Shi, Longxian Lv, Yi-Ling Du

**Affiliations:** 1 Institute of Pharmaceutical Biotechnology and The First Affiliated Hospital, Zhejiang University School of Medicine, 310058 Hangzhou, China; 2 School of Chemistry and Chemical Engineering, Henan Normal University, 453007 Xinxiang, China; 3 State Key Laboratory for Diagnosis and Treatment of Infectious Diseases, The First Affiliated Hospital, Zhejiang University, 310003 Hangzhou, China

**Keywords:** Biochemistry, Chemical biology, Microbiology

## Abstract

The heterocycle 1,2,3-triazole is among the most versatile chemical scaffolds and has been widely used in diverse fields. However, how nature creates this nitrogen-rich ring system remains unknown. Here, we report the biosynthetic route to the triazole-bearing antimetabolite 8-azaguanine. We reveal that its triazole moiety can be assembled through an enzymatic and non-enzymatic cascade, in which nitric oxide is used as a building block. These results expand our knowledge of the physiological role of nitric oxide synthase in building natural products with a nitrogen–nitrogen bond, and should also inspire the development of synthetic biology approaches for triazole production.

## Abstract (web summary)

How nature creates 1,2,3-triazole ring is a relevant question to N-N bond biosynthesis. Here, the authors report the biosynthetic route to the triazole-bearing antimetabolite 8-azaguanine and reveal that this can be assembled through an enzymatic and non-enzymatic cascade, in which nitric oxide is used as a building block.

## Introduction

**Fig. 1:**
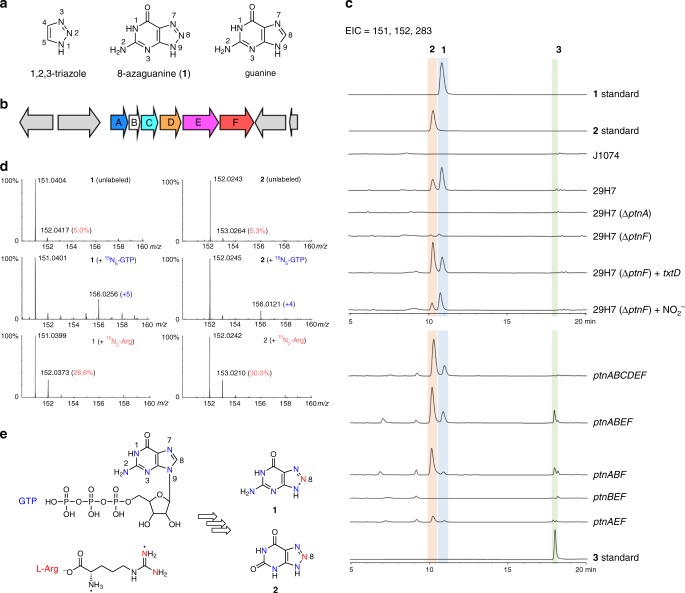
Biosynthetic investigation of 8-azaguanine (**1**). **a** The structures of 1,2,3-triazole, 8-azaguanine (**1**) and guanine. **b** The biosynthetic gene cluster (*ptn* cluster) of 8-azaguanine. **c** Heterologous expression and in vivo reconstitution of the **1** biosynthetic pathway in *S. albus* J1074. The extracted ion chromatograms (EIC) for **1** (*m*/*z* 151, [M−H]^−^), **2** (*m*/*z* 152, [M−H]^−^), **3** (*m*/*z* 283, [M−H]^−^) from LC-MS analysis were presented. Note: three independent tests were taken, the ratio of **1** to **2** varies from batches to batches, which could be attributed to the fluctuation of host metabolism, as **2** derives from **1** by a host enzyme (guanine deaminase). **d** LC-HR-ESI-MS analysis of 8-azaguanine (**1**) and 8-azaxanthine (**2**) from stable isotope-labeled precursors feeding experiments. **e** Isotope-labeling pattern of **1** and **2** based on results from (**d**).

Triazole-containing drugs exhibit remarkable diversity of chemical structures and biological activities including antimicrobial, antiviral, antitumor, anti-inflammatory effects^1^. The heterocycle 1,2,3-triazoles, which can be easily obtained by click chemistry, also have a wide range of applications in organic synthesis, bioconjugation, peptidomimetics and material science^2–4^. Despite the widespread occurrence of 1,2,3-trizaole scaffold in diverse fields, natural products bearing this nitrogen-rich heterocycle or its derivatives are scarce^5^. 8-azaguanine (**1**), also known as pathocidin, was originally reported as a synthetic guanine antagonist but later also isolated as a natural product from the culture broth of a *Streptomyces* strain (Fig. 1a)^6^. Structurally, **1** is a guanine analog with a rare naturally occurring 1,2,3-triazole fused with pyrimidine. As a member of purine and pyrimidine antimetabolites family, it displays a wide range of biological activities including anticancer, antiviral and antifungal effects^7^. The modes of action for the members (such as anticancer drugs 5-fluorouracil and 6-mercaptopurine) from this family are similar. As mimics of canonical nucleobases that serve as basic building unites of DNA and RNA, they can be transported into cells and converted to their respective nucleotides, which could then interfere with various cellular processes such as nucleic acid synthesis and protein synthesis^8^.

While the biosynthetic routes to natural products containing a N–N bond have received great interest and are starting to be revealed, how a triazole ring is assembled in nature remains unknown^9–23^. Elucidation of its biosynthetic mechanism could likely be helpful to the design of synthetic biology approaches for producing triazoles. We therefore are interested in the natural way of building the 1,2,3-triazolopyrimidine scaffold of **1**. Previous feeding experiments with ^14^C-labeling precursors revealed that the ^14^C carbon from [2-^14^C]-adenine or [2-^14^C]-guanine could be efficiently incorporated into **1**^24^. Furthermore, [8-^14^C]-guanine is not incorporated. Based on these results, it was speculated that **1** may derive from guanine by the substitution of a nitrogen atom for the C(8)–H group. However, the details of biosynthetic route to **1**, including the origin of the nitrogen, the timing of triazole ring formation, and the enzymes involved, have remained enigmatic.

In this study, by a combination of isotope-labeled precursors feeding experiments, in vivo gene knockout and reconstitution, as well as in vitro biochemistry, we unveil the biosynthetic genes responsible for the assembly of **1**. Moreover, we show that the triazolopyrimidine scaffold can be formed through an enzymatic and non-enzymatic cascade. Notably, we identified a bacterial nitric oxide synthase (NOS), which provides the nitrogen atom constituting the 1,2,3-trizole moiety. Our results thus expand the physiological roles of the widely occurring NOS in building N–N bond containing natural products.

## Results

### Isotope feeding study of 8-azaguanine biosynthesis

Although earlier feeding studies with ^14^C-labeled precursors have revealed the origin of carbon atoms in **1**, the origin of nitrogen atoms is still lacking^24^. We fed strain *Streptomyces albus* subsp*. pathocidicus* ATCC 14510 with the fully ^15^N-labeled guanosine triphosphate (^15^N_5_-GTP) at a final concentration of 1 mM, and analyzed the resulting **1** by LC-MS. The result revealed a new mass signal (*m*/*z* 158, [M + H]^+^) for **1**, with a +5 Da mass shift compared with unlabeled **1** (*m*/*z* 153, [M + H]^+^) (Supplementary Fig. 1), establishing that compound **1** retains all the five nitrogen atoms (N-1, N-2, N-3, N-7, N-9) from a single molecule of ^15^N_5_-GTP. Together with the earlier isotope feeding experiments, these results strongly indicate that construction of **1** involves the removal of the C(8)–H group of guanine, which could occur at the level of free nucleobase or in its nucleoside or nucleotide forms, and followed by re-cyclization with a nitrogen atom from an unknown source.

### Identification of the 8-azaguanine biosynthetic gene cluster

To unveil the biosynthetic route to this unusual triazole-containing molecule, we sequenced the genome of the **1** producer. The isotope-labeling pattern of **1** suggested that the triazole formation involves the cleavage of two carbon–nitrogen bonds (N-7/C-8 and C-8/N-9) and the formation of two new nitrogen–nitrogen bonds (N-7/N-8 and N-8/N9). Known enzymes that catalyze the release of the C(8)–H from guanine or its derivatives are mainly GTP cyclohydrolases (GCH) I and II (Supplementary Fig. 2)^25^. Despite that GCH I and GCH II share no sequence homology and convert GTP to different reaction products, they both mediate zinc-dependent hydrolytic opening of the imidazole ring of GTP, with the release of C(8)–H as formic acid. We initiated our search for genes related to **1** biosynthesis by scanning the genome for genes homologous to either GCH I or II, resulting in the identification of four candidate genes. Among them, *ptnA*, a putative GCH I gene, drew our attention (Fig. 1b). Analysis of its genomic context suggested that it is located in a putative gene cluster, named here as the *ptn* cluster, harboring genes encoding enzymes and a transporter related to nucleotide metabolism (Supplementary Table 1). These include a putative nucleosidase PtnC, a didomain protein PtnD consisting of N-terminal pyrophosphatase domain and C-terminal *N*-acetyltransferase domain, a purine efflux pump PtnE and a hypothetical protein PtnB. Interestingly, these genes appear to be in the same operon with a putative bacterial nitric oxide synthase (NOS) gene *ptnF*. To determine whether *ptnF*, and by extension the *ptnABCDEF* operon, was actively transcribed under the culture condition that **1** was produced, we performed reverse transcription polymerase chain reaction (RT-PCR) analysis on *ptnF*, revealing that the *ptn* cluster is transcriptionally active (Supplementary Fig. 3).

### In vivo genetic study of 8-azaguanine biosynthesis

To relate this putative *ptn* gene cluster to **1** biosynthesis, we first isolated cosmid 29H7 harboring the whole *ptn* cluster from a genomic library of the producing strain, and introduced 29H7 into *Streptomyces albus* J1074. Metabolic profiling by LC-MS analysis revealed that the resulting strain *S. albus* 29H7 produced a new compound with the same retention time and mass signal (*m*/*z* 153, [M + H]^+^) as that of the authentic **1** compound, whereas no corresponding peak is present in the culture of the parent strain *S. albus* J1074 (Fig. 1c). Furthermore, feeding ^15^N_5_-GTP to *S. albus* 29H7 also generated a strong +5 Da mass signal for this compound (from 151.0401 to 156.0256, [M−H]^−^) (Fig. 1d). Together, these data demonstrate that strain *S. albus* 29H7 produces **1**, and accordingly, cosmid 29H7 encodes the biosynthetic machinery directing **1** assembly. Besides compound **1**, *S. albus* 29H7 also produces an additional strain-specific molecule (**2**), whose mass signal increases by 4 Da (from 152.0245 to 156.0121, [M−H]^−^) when the strain was supplemented with ^15^N_5_-GTP (Fig. 1c, d). This labeling pattern indicated that compound **2** retained four nitrogen atoms from GTP. We identified **2** as 8-azaxanthine by comparing its HPLC retention time and ^1^H NMR spectrum with that of an authentic compound (Fig. 1e and Supplementary Fig. 4). Compound **2** was previously shown to be a host-processed metabolite of **1**^7^. The conversion of **1** to the non-toxic compound **2**, mediated by guanine deaminase, is also known as the resistance mechanism of **1**. To confirm that **2** was a metabolite of **1** by enzyme(s) from the host, we fed **1** to *S. albus* J1074 at 24 h and observed the full conversion of **1** to **2** at 96 h (Supplementary Fig. 5).

The co-occurrence of the putative NOS gene *ptnF* in an operon with the GCH I-like gene *ptnA* indicates the potential involvement of nitric oxide (NO) in the biosynthesis of **1**. We next explore whether the N-8 nitrogen atom of **1** and **2** originate from NO. NOS is known to convert l-arginine to l-citrulline and NO, with *N*^*G*^-hydroxyl-l-arginine as an catalytic intermediate, and the nitrogen atom of NO derives from a guanidino nitrogen of l-arginine^26,27^. We next fed strain *S. albus* 29H7 with l-[guanidino-^15^N_2_]-arginine at a final concentration of 1 mM and analyzed the resulting **1** and **2** by LC-HR-ESI-MS. We found that the relative intensity of +1 Da peak increased from 5.0 to 28.6% for **1** and 5.3 to 30.0% for **2** when compared with unlabeled compounds, suggesting that a guanidino nitrogen atom of l-[guanidino-^15^N_2_]-arginine is incorporated into **1** and **2** (Fig. 1d, e). To further link *ptnA* and *ptnF* to the biosynthesis of **1**, we generated strain *S. albus* 29H7(Δ*ptnA*) and 29H7(Δ*ptnF*), which carries an engineered cosmid 29H7 in which the *ptnA* and *ptnF* gene was inactivated, respectively (Supplementary Fig. 6). Subsequent LC-MS analysis revealed that the production of **1**/**2** is completely abolished in *S. albus* 29H7(Δ*ptnA*), whereas only trace amount of **1**/**2** is detected in *S. albus* 29H7(Δ*ptnF*). These data demonstrate that *ptnA* and *ptnF*, and by extension, the *ptn* cluster are responsible for the biosynthesis of **1**. Next, we interrogated the role of PtnF through genetic and chemical complementation. We found that providing strain *S. albus* 29H7(Δ*ptnF*) with a copy of gene *txtD*, which encodes the characterized NOS participating in l-tryptophan nitration in the biosynthesis of thaxtomin phytotoxins^28,29^, restored the yield of **1**/**2** to the level that is comparable to that of *S. albus* 29H7. Moreover, supplementing strain *S. albus* 29H7(Δ*ptnF*) with sodium nitrite (at a final concentration of 10 mM) also increased the titer of **1**/**2** (Fig. 1c).

**Fig. 2:**
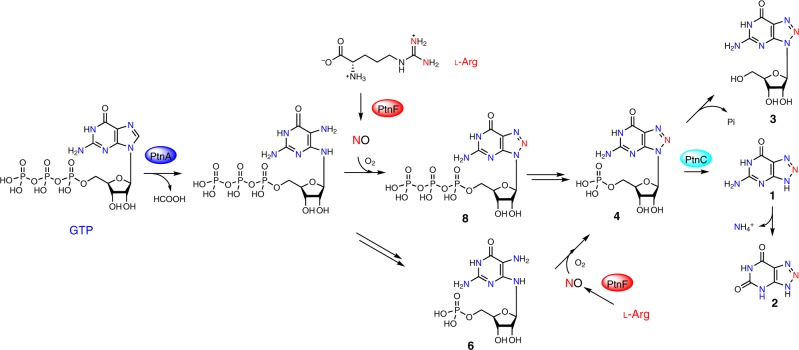
Proposed biosynthetic route to 8-azaguanine (**1**) based on this study. The GCH I-like enzyme PtnA is proposed to catalyze the hydrolytic cleavage of C8 of GTP. The resulting product 2,5-diaminopyrimidine triphosphate could combine with a NO-derived reactive species to afford 8-azaguanosine triphosphate (**8**), and followed by pyrophosphate release by host enzyme(s). Alternatively, the compound 2,5-diaminopyrimidine triphosphate could first undergo pyrophosphate release and then followed by triazole formation to afford **4**.

To determine the minimal set of biosynthetic genes required for **1** assembly and provide insights into the roles of *ptn* genes, we undertook an in vivo reconstitution strategy. Engineered *S. albus* strains harboring different combinations of *ptn* genes were generated and subjected to metabolic profiling by LC-MS (Supplementary Fig. 7 and Fig. 1c). The results showed that *S. albus* strain carrying the *ptnABCDEF* operon produces **1**/**2** at a titer comparable to that of *S. albus* 29H7, indicating that this operon is sufficient for **1**/**2** biosynthesis in *S. albus*. Moreover, both strain *S. albus ptnABEF* and *ptnABF* also generate significant amounts of **1**/**2** but accumulate a new compound **3**, suggesting that deletion of either the putative nucleosidase gene *ptnC* or the pyrophosphatase-domain-containing protein gene *ptnD* is responsible for the production of **3**. We then constructed *S. albus* 29H7(Δ*ptnC*) and *S. albus* 29H7(Δ*ptnD*) and subjected them for LC-MS analysis, showing that **3** only accumulates in *S. albus* 29H7(Δ*ptnC*) (Supplementary Fig. 8). Next, we sought to characterize compound **3**. We first fed strain *S. albus ptnABEF* with l-[guanidino-^15^N_2_]-arginine and analyzed the resulting **3** by LC-MS. The result revealed the enrichment of the +1 Da isotope peak, confirming that **3** also arise from the biosynthetic pathway of **1** (Supplementary Fig. 9). We then isolated **3** from a scale-up culture (2.8 L) of *S. albus ptnABEF* and identified **3** to be 1,2,3-triazole nucleoside 8-azaguanosine, based on NMR analysis and chemical degradation experiment (Fig. 2 and Supplementary Fig. 9). Strain *S. albus ptnAEF* produces **1**/**2**/**3** with decreased titer, indicating that PtnB, which has no characterized homolog, could be involved but not essential for the biosynthesis of **1**. The observation that no **1**/**2** was detected in the culture broth of *S. albus ptnBEF* again demonstrated that PtnA is indispensable.

### PtnF is a bacterial nitric oxide synthase

**Fig. 3:**
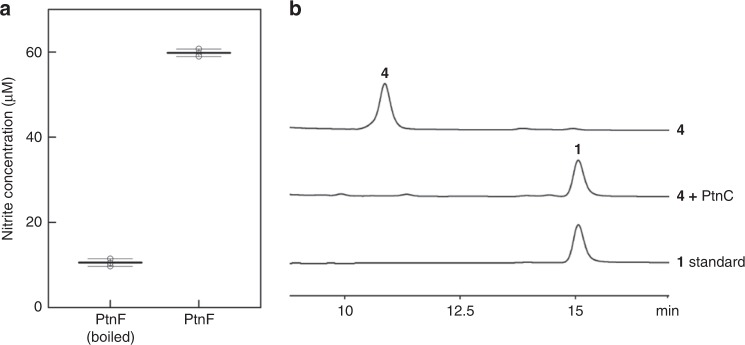
In vitro biochemical assays of PtnF and PtnC. **a** Detection of nitrite production in biochemical assays of PtnF by Griess reagent. Three independent tests were taken. **b** Detection of **1** production in biochemical assays of PtnC by HPLC (detection wavelength = 254 nm).

Having establishing the biosynthetic origin of **1** and the necessity of each *ptn* gene for **1** biosynthesis in *S. albus*, we next interrogated the role of Pat enzymes in vitro. Our above feeding experiments and in vivo genetic studies have suggested that PtnF could be a bacterial NOS, providing NO as the nitrogen source of N-8 atom in **1**. Bioinformatic analysis revealed that PtnF shares 63% sequence identity with TxtD, and the active residues of NOS are highly conserved in PtnF (Supplementary Fig. 10a). To further ascertain its role as a NOS, we performed in vitro biochemical assay of PtnF by examining its ability to convert *N*^*G*^-hydroxyl-l-arginine to NO in the presence of hydrogen peroxide, which is an assay that has been widely used for in vitro characterization of NOS^28^. We expressed and purified His_6_-PtnF protein from the *E. coli* host, and incubated it with *N*^*G*^-hydroxyl-l-arginine and hydrogen peroxide. After incubation for 15 min at room temperature and quenching with catalase, nitrite was successfully detected in the reaction mixture by using the Griess reagent (Fig. 3a and Supplementary Fig. 10b, c). All together, these results ambiguously established PtnF as a bacterial NOS.

### PtnC is an 8-azaguanosine monophosphate nucleosidase

We next interrogated the role of PtnC in **1** biosynthesis. The observation that compound **3** only accumulates in the engineered *S. albus* strains lacking *ptnC* indicated that **3** is an intermediate or a shunt product from the biosynthetic pathway, instead of a host-modified metabolite of **1**. Sequence analysis of PtnC revealed that it displays limited sequence homology to nucleosidases such as cytokinin riboside 5′-monophosphate phosphoribohydrolases and the central catalytic domain of *E. coli* nucleosidase PpnN, both of which convert nucleoside monophosphates to ribose-5′-phosphate and the corresponding nucleobases (Supplementary Fig. 11a)^30,31^. Moreover, although there is sequence variation in the putative substrate binding motif, residues (Arg95 and Glu118) potentially involved in catalysis are conserved in PtnC. Based on the above in silico and genetic data, we speculated that PtnC cleaves the *N*-glycosidic bond of 8-azaguanosine 5′-monophosphate (**4**) to release **1**, and compound **3** could derive from **4** through dephosphorylation by host nonspecific phosphatases (Fig. 2). To test this hypothesis, we synthesized **4** by a chemoenzymatic method and incubated it with purified His_6_-PtnC (Supplementary Fig. 11b). HPLC analysis of reaction mixture revealed the full conversion of **4** to **1**, whereas no *N*-glycosidic bond cleavage activity was detected against guanosine monophosphate (GMP), 8-azaguanosine or 2,5-diamino-6-ribosylamino-4(3*H*)-pyrimidinone 5′-phosphate (see below) (Fig. 3b and Supplementary Fig. 11c). Together, these results demonstrate that PtnC is an 8-azaguanosine monophosphate nucleosidase, and the conversion of **4** to **1** could be the last step in **1** biosynthesis.

### Non-enzymatic assembly of the triazole ring

**Fig. 4:**
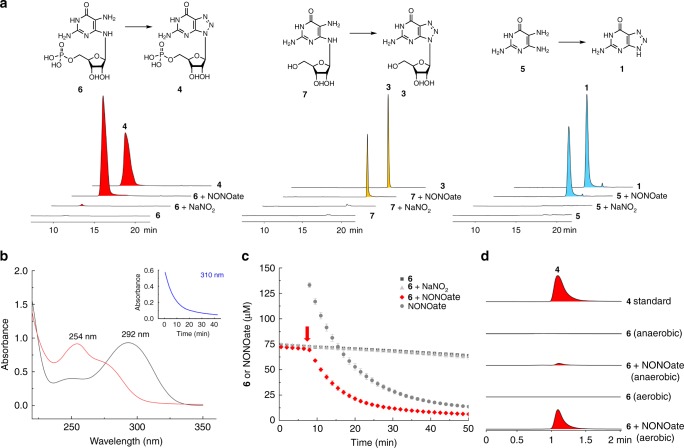
Non-enzymatic triazole assembly. **a** LC-MS analysis of the reaction mixtures of **5**, **6**, or **7** with NaNO_2_ or DEA NONOate. The EICs for **4** (*m*/*z* 363, [M−H]^−^), **3** (*m*/*z* 283, [M−H]^−^), and **1** (*m*/*z* 151, [M−H]^−^) are presented. Compound **5**, **6**, or **7** (0.5 mM) are incubated with DEA NONOate (1 mM) or sodium nitrite (1 mM) at 30 °C for 1 h before LC-MS analysis. Authentic **1**, **3**, **4** were used as analytical standards. **b** Change of **6** (75 μM, black line) absorption spectra upon the addition of NONOate (150 μM) in 50 mM phosphate buffer (pH 7.4) at 25 °C. The red trace was collected 1 h after the addition of NONOate. The absorbance at 310 nm was monitored with a 1.5-min time interval (as shown in the insert figure). **c** Time-course study of the reaction of **6** (75 μM) with NONOate (150 μM) or nitrite (150 μM). Red arrow indicates the time point when NONOate or nitrite was added. Values are means of three independent experiments ± SD. Source data are provided as a Source Data file. **d** LC-MS analysis of the reaction mixtures of **6** (75 μM) and NONOate (1 mM) prepared anaerobically and aerobically. The EICs for **4** (*m*/*z* 363, [M−H]^−^) are presented. Three independent tests were taken.

Establishment of nitric oxide and compound **4** as biosynthetic precursors of **1** indicates that the triazole formation is prior to the *N*-glycosidic bond cleavage. Triazole formation between NO-related reactive nitrogen species and compounds with vicinal diamines is reminiscent of a widely-used method for in situ NO detection and imaging, which involves non-enzymatic capture of NO by synthetic chemicals such as 4,5-diaminofluorescein to generate fluorescent triazofluoresceins^32^. Considering that only the GCH I-like protein PtnA is indispensable for **1** production in our genetic studies, we next explore whether the triazolopyrimidine scaffold could be assembled non-enzymatically. We envisaged that the direct precursor of the triazolopyrimidine structure is mostly likely the nucleotide or nucleoside derivative of 4-hydroxy-2,5,6-triaminopyrimidine (**5**) (Fig. 4a). We prepared 2,5-diamino-6-ribosylamino-4(3*H*)-pyrimidinone 5′-phosphate (**6**) and 2,5-diamino-6-ribosylamino-4(3*H*)-pyrimidinone (**7**) as the potential precursors (or their structural analogs). We then separately incubated **5**, **6**, **7** with NO donor diethylamine (DEA) NONOate or sodium nitrite in the buffer with a pH value of 7.5, which was chosen based on a time-course studies of pH values and **1**/**2** production during fermentation (Supplementary Fig. 12). LC-MS analysis demonstrated that all three substrates (**5**, **6**, **7**) were efficiently converted to the corresponding triazole products (**1**, **4**, **3**) in the reactions with NONOate, but only trace or none of these products could be observed when incubated with the same concentration (1 mM) of sodium nitrite (Fig. 4a). Using **6** as a model substrate, we then monitored the reaction process spectroscopically. We found that the absorption spectrum of **6** changed to a trace that is very similar to that of synthetic **4** after 1 h of incubation with two equivalents of NONOate, indicating the full conversion of **6** to **4** (Fig. 4b and Supplementary Fig. 13). Time-course study of **6** consumption revealed that **6** rapidly converts to **4** upon the addition of NONOate, whereas nitrite did not consume **6** at the same concentration (150 μM) (Fig. 4c and Supplementary Fig. 14). Moreover, the consumption rate of **6** increases as the concentration of NONOate increases, and the triazole product could still be observed at a low level of NONOate (20 μM) (Supplementary Fig. 15). The time-course of NONOate decomposition was also studied under the same condition, revealing a half-life of ~10 min for DEA NONOate (Fig. 4c and Supplementary Fig. 14).

As NO rapidly undergoes stepwise oxidation to nitrite in aerobic condition, we next interrogate the role of molecule oxygen in this non-enzymatic transformation, we incubated **6** with NONOate in an anaerobic glovebox, and observed **4** production in very low yield (~8% relative to that of the control assay conducted aerobically) (Fig. 4d). This result suggests that it is the oxidized species of NO that directly participating in triazole production. Considering that nitrite did not cause triazole formation under this condition, this oxidized species is likely to be dinitrogen trioxide (N_2_O_3_), an autoxidation product of NO on the way of its stepwise oxidation to nitrite (see discussion)^33^. We also interrogate the effect of nitrite concentration on triazole formation, and found that nitrite could also promote triazole formation at higher concentrations (Supplementary Fig. 16a). Moreover, we observed that the conversion of **6** to **4** could occur at all pH values (6.0, 7.0, 8.0) we tested (Supplementary Fig. 16b). Taken together, these results suggested that NO-derived reactive nitrogen species might be responsible for the assembly of the triazole moiety in **1**.

### The GCH I-like protein PtnA and early biosynthetic steps

Compared with later biosynthetic transformations, the early steps in the biosynthetic pathway are currently less clear. Sequence analysis and homology modeling of PtnA revealed that it is a homolog of GCH I, which typically converts GTP to dihydroneopterin triphosphate through a multistep reaction including the zinc-assisted hydrolytic release of C-8 atom, as well as the subsequent rearrangement followed by ring closure (Supplementary Fig. 2)^25^. PtnA has highly conserved residues putatively involved in zinc coordination (Cys90, His92, His93, Cys161) and GTP binding, which occurs through the hydrogen bond interactions with the guanine moiety (Glu132) and triphosphate group (Arg47, Ser115, Lys116, Arg165) of GTP (Supplementary Fig. 17a). These data strongly suggest that PtnA catalyzes a GCH I-like reaction. However, PtnA only has ~40% sequence identity with typical housekeeping GCH I of *Streptomyces* origin, and forms a separate branch different from canonical GCH I from various organisms in phylogenetic analysis, suggesting a specialized metabolic role for PtnA (Supplementary Fig. 17b). As our attempt to obtain soluble PtnA protein failed, we use in vivo reconstitution approach to explore the role of the key residue His92, the counterpart of which participates in both catalysis and zinc binding in *E. coli* GCH I FolE^34^. In line with the in silico analysis, an engineered *S. albus* strain harboring a H92A variant of *ptnA* gene together with genes *ptnBEF* failed to generate detectable level of **1**/**2**, demonstrating the essential role of His92 in PtnA-catalyzed reaction (Supplementary Fig. 17c). In view of the chemical structures of **1** and **4** in which no carbon-skeleton rearrangement seems to be required, it is likely that PtnA catalyzes a zinc-dependent hydrolytic cleavage of C8, as occurs in canonical GCH I-catalyzed reaction, but might be deficient in promoting the subsequent rearrangement (Fig. 2). The resulting product 2,5-diaminopyrimidine triphosphate could then combine with a NO-derived reactive species to afford 8-azaguanosine triphosphate (**8**), and followed by pyrophosphate release by enzymes from the host purine metabolic pathways to give **4**. Alternatively, the compound 2,5-diaminopyrimidine triphosphate could first undergo the release of pyrophosphate and followed by triazole formation to afford **4**.

To probe whether the product from PtnA-catalyzed reaction or other ribosyl derivatives of **5** could accumulate in engineered *S. albus* strains deficient in NO production, the culture supernatants from selected strains were subjected to LC-MS analysis, with or without the treatment of DEA NONOate (Supplementary Fig. 18). Synthetic **8** (and **3**, **4**) were prepared and used as analytical standards (Supplementary Fig. 13). However, none of these compounds or derivatives was detected. It is likely that those potential pathway intermediate(s) could be rapidly metabolized by host enzymes, or decomposed in culture broth, as it has been known that ribosyl derivatives of **5** are highly reactive, and has a half-life of only a few hours^35^.

## Discussion

Natural products containing a N–N bond is a relatively small group of molecules with diversified chemical structures and biological activities^36^. To connect two nitrogen atoms together, nature has devised different strategies. The most frequently used precursors for N–N bond formation are hydroxylamines (such as l-*N*^5^-OH-Ornthine in piperazate pathway^9^, *N*-isobutylhydrdoxylamine in valanimycin pathway^37^, and l-*N*^6^-OH-lysine in the pathways of s56-p1^14^, pyrazomycin^16^ and formycin^17,38^) and nitric acid, which derives from the α-amine of l-aspartic acid by the enzyme pair CreE and CreD^10^. The flavin monooxygenase CreE and nitrosuccinate lyase CreD were first characterized in the cremeomycin pathway, and their homologs were subsequently identified in the pathways of fosfazinomycin, and kinamycin, triacsins and alanosine^15,21–23^.

Nitric oxide has diverse physiological functions including acting as an ubiquitous messenger molecule in diverse cellular processes^27,39–41^. Canonical heme-dependent nitric oxide synthases (NOS) have been found in a variety of organisms ranging from bacteria to plants and mammals. Despite the different domain organizations, the reaction they catalyzed are highly conserved. In the field of natural product biosynthesis, NOS was previously only related to nitration of aromatic precursors, such as l-4-nitrotryptophan and l-3-nitrotyrosine^28,29,42,43^. In this study, we revealed the involvement of a bacterial NOS in an enzymatic and non-enzymatic cascade leading to the formation of a rare 1,2,3-triazolopyrimidine scaffold. We propose that this reaction proceeds through a NO-initiated nitrosative cyclization mechanism in vivo, with the intermediacy of a *N*-nitrosamine species (Supplementary Fig. 19). The O_2_-dependence of this non-enzymatic triazole formation suggests that the reactive nitrogen species directly participating in *N*-nitrosation is most likely to be N_2_O_3_ (or nitrosonium cation NO^+^), which is an autoxidation product of NO and has potential nitrosating activity^33^. In the case of nitrite (NO_2_^−^), it has been known that NO_2_^−^ can also convert to N_2_O_3_/NO^+^ under acidic conditions. The observation that a much higher concentration of NO_2_^−^ (compared with that of NO) was required to promote triazole formation is not unexpected, as N_2_O_3_/NO^+^ formation from NO_2_^−^ should be much less efficient in the buffer (pH 7.5) we used. The successfully chemical complementation of the NOS mutant with NaNO_2_ could be due to the conversion of NO_2_^−^ to N_2_O_3_/NO^+^ in vivo. Alternatively, NO_2_^−^ could also be first reduced to NO by the host nitrite reductase (Supplementary Table 3). Considering that NO_2_^−^ was only detected at low micromole level in *S. albus* strains (Supplementary Fig. 20), the biosynthesis of **1** via the in vivo non-enzymatic process is expected to proceed through NO-derived nitrosation.

Although we have demonstrated that the 1,2,3-triazolopyrimidine scaffold of **1** can be efficiently assembled non-enzymatically, we cannot exclude the possibility that an enzymatic process, mediated by either a dedicated enzyme or a non-specialized one, is also involved in this transformation in vivo. The results from our genetic studies showed that the *ptnABCDEF* operon alone is sufficient for **1** production, suggesting that the potential enzyme for triazole formation could be either encoded by this operon, or located elsewhere in the genome of *S. albus* (and the original producer). In the *ptnABCDEF* operon, the function of *ptnB* has not been assigned yet. PtnB is a small protein without any close homologs or predicted functional domains. Spectroscopic analysis of as-purified PtnB indicated that PtnB is an iron-binding metalloprotein (Supplementary Fig. 21a, b). It is worth to mention that metalloproteins have been linked to N–N bond formation in the biosynthetic pathways of molecules such as piperazate^9^, streptozotocin^12^, s56-p1^14^, and pyrazomycin^16^. The observation that strain *S. albus ptnAEF* produces less amount of **1** compared with *S. albus ptnABEF* suggests that PtnB might be a candidate enzyme for triazole formation. However, we did not observe any such activity when incubating PtnB with **6** in the presence of NONOate, both aerobically and anaerobically (Supplementary Fig. 21). We are aware that PtnB might use an alternative substrate, such as the diphosphate or triphosphate derivatives of **6**, however, we could not obtain them through chemical synthesis due to their chemical instability. It is also possible that the purified PtnB might be inactive due to the lack of additional cofactors or protein partners. Another possible role for PtnB is that it may assist one of the biosynthetic enzymes (such as PtnA) to perform their function. The detail role of PtnB waits further investigation.

In conclusion, we reported the biosynthetic gene cluster of a 1,2,3-triazole-bearing natural product, and we showed that nitric oxide is used as a biosynthetic precursor for the unique 1,2,3-triazolopyrimidine scaffold of 8-azaguanine. Our results expanded the physiological role of canonical nitric oxide synthase in building N–N-bond-containing molecule, and may inspire the development of synthetic biology approaches for triazole production.

## Methods

### General methods

DNA primers were purchased from Sangon Biotech and Tsingke Biological Technology. Reagents were purchased from Sigma-Aldrich, Thermo Fisher Scientific, Cambridge Isotope Laboratories, New England BioLabs, Bio Basic Inc. DNA manipulations in *Escherichia coli* and *Streptomyces* strains were carried out according to standard procedures^44,45^. *Streptomyces* and its mutant strains were normally maintained on MS (2% mannitol, 2% soy flour, 2% agar) agar or ISP2 agar (1% malt extract, 0.4% yeast extract, 0.4% glucose, 2% agar). Ampicillin (100 μg mL^−1^), apramycin (50 μg mL^−1^), kanamycin (50 μg mL^−1^), spectinomycin (50 μg mL^−1^), and nalidixic acid (25 µg mL^−1^) were used for selection of recombinant *E. coli* and *Streptomyces* strains. Genome sequencing and in silico analysis of genome sequence assembled 7.28 megabase pairs of nonredundant sequences over 181 contigs with 366-fold read coverage (Note: the genome of the same strain *Streptomyces pathocidini* NRRL B-24287 was also sequenced by another group and deposited in Genbank under the accession number NZ_LIQY00000000.1). Cosmid 29H7 containing the whole 8-azaguanine biosynthetic gene cluster, from the genomic library of *Streptomyces albus* subsp*. pathocidicus* ATCC 14510, was obtained by library screening with PCR using primers targeting *ptnA* and *ptnF* genes (Supplementary Table 2).

### RNA isolation, RT-PCR

The total RNA of *Streptomyces albus* subsp*. pathocidicus* ATCC 14510 was isolated from the strain grown in YEME medium (0.5% peptone, 0.3% yeast extract, 1% glucose, 0.3% malt extract) at 36 h. RNA was prepared with Trizol (Sangon) according to the manufacturer’s instructions. Genomic DNA was removed by RNase-free DNase I (Takara), and the RNA integrity was checked by agarose gel electrophoresis, and its concentration was determined by using Nanodrop (Thermo Fisher Scientific). Two-step RT-PCR was performed: cDNA was first made from 1 µg total RNA using a Primescript cDNA Synthesis Kit (Takara) based on the manufacturer’s manual, and cDNA was amplified by Taq DNA polymerase (Bio Basic, Inc.) with *ptnF* primers (Supplementary Table 2). The PCR conditions consisted of one cycle of denaturation at 94 °C (2 min), followed by 30 cycles of 94 °C (30 s), 58 °C (30 s), and 72 °C (60 s), with one extension cycle at 72 °C (5 min).

### Heterologous expression and engineering of the *ptn* cluster

Cosmid 29H7 was passaged through non-methylating host *E. coli* ET12567/pUZ8002 before being introduced into *Streptomyces albus* J1074 via conjugation. Apramycin-resistant exconjugates were confirmed for site-specific integration by PCR, resulting in strain *S. albus* 29H7. For construction of *S. albus* 29H7(Δ*ptnF*) harboring an in-frame deleteion of the *ptnF* gene*, ptnF* was first inactivated in 29H7 by insertion of an *aadA* gene (spectinomycin resistant gene) amplified from pHY773 into its coding region, using the λ-RED-mediated PCR targeting method^46^. The targeted plasmid was then transformed into *E. coli* DHα/BT340 in order to excise the *aadA* cassette. The resulting plasmid was designated 29H7(Δ*ptnF*). After confirmation with restriction and PCR analysis, 29H7(Δ*ptnF*) was passaged through *E. coli* ET12567/pUZ8002 before being introduced via conjugation into *S. albus* J1074 to afford *S. albus* 29H7(Δ*ptnF*). Apramycin-resistant colonies were confirmed for integration and gene inactivation by PCR with the primer pairs for *ptnF* (Supplementary Table 2). The construction of strains *S. albus* 29H7(Δ*ptnC*) and *S. albus* 29H7(Δ*ptnD*) were carried out similarly as above. For the construction of *S. albus* 29H7(Δ*ptnA*), an inactivation cassette consisting of aadA-ermE*p was used to replace part of this coding region.

For in vivo reconstitution, different combinations of *ptn* genes were cloned and arranged in the integrative vector pYLD20^47^ and driven by a strong constitutive promoter ermE*p (Supplementary Fig. 7). After confirmation with restriction and PCR analysis, these vectors were passaged through *E. coli* ET12567/pUZ8002 before being introduced via conjugation into *S. albus* J1074 to give corresponding engineered *S. albus* strains. Genetic complementation of 29H7(Δ*ptnF*) with the characterized NOS gene *txtD* was performed similarly as described above. Gene *txtD* and the DNA fragment containing *ptnA*(H92A)-*ptnB* were chemical synthesized at Sangon Biotech (Shanghai, China).

### Metabolic analysis for the *Streptomyces* strains

Spore suspensions of *Streptomyces* strains were used to inoculate 250-mL flasks containing 50 mL of tryptic soy broth (BD) medium, which were incubated with shaking for 18 h at 30 °C. 2.5 ml samples of the seed cultures were then used to inoculate 250 mL flasks containing 50 mL of the YEME medium (for *Streptomyces albus* subsp*. pathocidicus* ATCC 14510) or modified R5 medium [(g L^−1^): K_2_SO_4_ (0.25), MgCl_2_·6H_2_O (0.25), glucose (10.0), casamino acid (0.1), yeast extract (5.0), CaCO_3_ (2.0), trace elements^45^ 2 mL L^−1^] (for engineered *S. albus* J1074 strains) and these were incubated with shaking at 30 °C. In the case of isotope-labeled precursors feeding experiments, ^15^N-labeled guanosine triphosphate (^15^N_5_-GTP) or l-[guanidino-^15^N_2_]-arginine were added at final concentrations of 1 mM at 24 h. After another 72 h, cultures were centrifugated at 12,000 rpm for 5 min to remove the mycelium. The supernatants were then subjected to ultrafiltration using Amicon Ultra-0.5 mL centrifugal filters (Millipore, 10,000 MWCO) before HPLC or LC-MS analysis.

Analytic HPLC analysis was carried out with an Agilent 1260 HPLC apparatus using an Agilent Elipse XDB-C18 column (5 μm, 4.6 mm ID × 250 mm). Elution was performed at 0.6 mL min^−1^ with a mobile-phase mixture consisting of a linear gradient of water and acetonitrile ((v/v): 98:2, 0–10 min; 50:50, 10–20 min; 5:95, 20–25 min), both of which contain 0.05% (v/v) formic acid. Authentic compounds of **1** and **2** (Sigma-Aldrich) were used as a control (detection wavelength: 254 nm). LC-MS was performed under the same conditions on the Agilent 6125 LCMS system operated in positive mode or negative mode. LC-HR-ESI-MS was performed similarly on waters UPLC coupled with an AB TripleTOF 5600+ mass spectrometer system.

For isolation of compound **3** produced by strain *S. albus ptnABEF*, Amberlite XAD 16N beads (15 g L^−1^) was added to the 2.8 L culture supernatant, and mixed with shaking (120 rpm) at room temperature for 2 h. The filtrate collected from the above mixture was then concentrated to 200 mL and deproteinated by precipitation with 4 volumes of ethyl alcohol. After concentrated under vaccum, the residue was fractionated on Sephadex LH-20 with MeOH:H_2_O (1:1) elution. Metabolites of interest, tracked by analytical HPLC, were purified from these fractions by reversed-phase semi-preparative HPLC (YMC-Triart C18, 5 µm, 10 mm ID × 250 mm). The ^1^H- and ^13^C-and 2D NMR spectra were recorded on a Bruker AV-600 MHz spectrometer using D_2_O as the solvent.

The concentration of nitrite in culture broths or in assay mixtures was determined with a nitrite detection kit (Cat. NO: S0021 from Beyotime Biotech), which is based on the reaction of nitrite with the Griess reagents. Culture supernatants of *S. albus* strains were obtained after removal of mycelium by centrifugation. The assays were performed according to the manufacturer’s instructions.

### Protein expression and purification

The coding regions of *ptnA*, *ptnF*, *ptnC*, and *ptnB* were amplified by PCR from cosmid 29H7 and cloned into vector pET22b or pET28a to afford expression plasmids pET22b-*ptnA*, pET22b-*ptnB*, pET28a-*ptnF*, pET28a-*ptnC*, which were then transformed into *E. coli* BL21 (DE3) for expression. To prepare starting culture, cells harboring corresponding pET vectors were grown overnight in Luria-Bertani (LB) broth with 50 µg mL^−1^ kanamycin (for pET28a-*ptnF* and pET28a-*ptnC*) or 100 µg mL^−1^ ampicillin (for pET22b-*ptnA* and pET22b-*ptnB*) at 37 °C and 200 rpm. A starting culture (2.5 mL) was then used to inoculate LB broth (750 mL) containing appropriate antibiotics. The culture was grown at 37 °C and 200 rpm to an optical density of 0.6 at 600 nm, and then cooled to 16 °C. Isopropyl β-d-1-thiogalactopyranoside (final concentration 0.1 mM) was added to induce overproduction of the protein.

After 20 h of further incubation, the cells were harvested and resuspended in lysis buffer (300 mM NaCl, 50 mM Tris-HCl, 10 mM imidazole, pH 8.0) and then disrupted by sonication, before the recovery of supernatant by centrifugation (13,000 × *g* for 40 min). His-tagged protein was separated using nickel-nitrilotriacetic acid (Ni-NTA) resin. After being washed with washing buffer (300 mM NaCl, 50 mM Tris-HCl, 50 mM imidazole, pH 8.0), protein was eluted with elution buffer (300 mM NaCl, 50 mM Tris-HCl, 250 mM imidazole, pH 8.0). Purified protein-containing fractions were confirmed by SDS–PAGE analysis, and then dialyzed into the storage buffer (50 mM Tris-HCl, 100 mM NaCl, 10% glycerol, pH 8.0), concentrated and stored at −80 °C for further use. Protein concentration was determined by using the Bradford protein assay (Bio Basic Inc).

### In vitro biochemical assays

For the in vitro assay of PtnF, the reaction mixture (50 µl) contained 5 µM PtnF, 0.5 mM *N*^*G*^-hydroxyl-l-arginine (Sigma-Aldrich), H_2_O_2_ (40 mM) in 50 mM HEPES buffer (pH 7.5). The reaction mixture was incubated for 15 min at room temperature and quenching with 200 Units of catalase (Sigma-Aldrich), followed by nitrite detection using Griess reagent I and II as described above. Heat-inactivated PtnF was used as a negative control.

For the in vitro assay of PtnC, 8-azaguanosine 5′-monophosphate (**4**) was first generated in situ by a chemoenzymatic method, performed as follows: compound 2,5-diamino-6-ribosylamino-4(3 *H*)-pyrimidinone 5′-phosphate (**6**) was first enzymatically prepared with the *E. coli* GTP cyclohydrolase II RibA in a reaction mixture (200 µl) consisting of 200 µg RibA (prepared similarly as that for PtnF), 5 mM GTP, 1 mM MgCl_2_, 5 mM DTT, in 50 mM Tris buffer (pH 7.0). The reaction mixture was incubated for 1 h at room temperature and deproteinated by ultrafiltration using Amicon Ultra-0.5 mL centrifugal filters (Millipore, 10,000 MWCO). Sodium nitrite (at a final concentration of 10 mM) was then added to the above filtrate and followed by shaking (200 rpm) at 25 °C for another 1 h to give **4**, which was used without further purification. Purified PtnC (final concentration of 20 µM) was added to the above solution and incubated with **4** at 30 °C for 1 h before HPLC analysis. HPLC analysis was conducted using a Luna C18(2), 5 μm, 4.6 mm ID × 250 mm column (Phenomenex), with the same detection wavelength and elution condition as described for the above metabolic analysis experiments.

### Enzymatic synthesis of compounds **6** and **7**

For preparation of **6**, a reaction mixture (5 mL) consisting of 5 mg *E. coli* RibA, 5 mM GTP, 1 mM MgCl_2_, 5 mM DTT in 50 mM Tris buffer (pH 8.5) was incubated at room temperature for 1 h. After deproteinated by ultrafiltration, compound **6** in the filtrate was purified with reversed-phase semi-preparative HPLC (YMC-Triart C18, 5 µm, 10 mm ID × 250 mm) eluting with 2% acetonitrile. The fractions containing **6** were immediately frozen at −80 °C and followed by lyophilization. Compound **7** was prepared similarly as **6**, except that a step of dephosphorylation by calf intestinal alkaline phosphatase (CIAP) was added before ultrafiltration.

### In vitro non-enzymatic triazole formation

For the non-enzymatic reactions of compound **5** (available at Sigma-Aldrich), **6**, **7** with the nitric oxide donor diethylamine (DEA) NONOate or sodium nitrite, reaction mixtures (50 µL) contained 0.5 mM **5**/**6**/**7** and different concentrations of DEA NONOate or sodium nitrite in 50 mM Tris-HCl buffer with different pH values were shaken (200 rpm) at 30 °C for 1 h. All the mixtures were then frozen at −80 °C, and thawed right before LC-MS analysis using a Phenomenex Luna C18(2) column (5 μm, 4.6 mm ID × 250 mm), using the same detection wavelength and elution condition as described for the in vitro enzyme assays.

### UV-vis spectroscopic analysis

For UV-vis spectroscopic monitoring of in vitro non-enzymatic triazole formation, assays were performed in a final volume of 600 µL phosphate buffer (50 mM, pH 7.4) in a 0.7 mL-quartz cuvette at 25 °C. The compound **6** (75 μM) and buffer solution were equilibrated for ~8 min before the addition of DEA NONOate (0, 20, 50, 75, 150, and 300 μM) or nitrite (150 μM) to initiate the reaction. The reaction mixtures were scanned (from 350 to 220 nm) at a 1.5-min time interval for a total of 60 min. Spectra were recorded using a Shimadzu UV-2600 spectrophotometer. The consumption of **6** was followed by monitoring the absorbance at 310 mm. A standard curve of **6** was made under the similar condition (Supplementary Fig. 14a).

For the investigation of the decomposition process of DEA NONOate under the above reaction condition, a stock solution of DEA NONOate (50 mM) prepared in 10 mM of NaOH solution was added to a quartz cuvette containing 600 µL phosphate buffer (50 mM, pH 7.4, 25 °C). The decomposition of DEA NONOate (final concentration: 150 μM) was monitored spectroscopically at 248 nm (Supplementary Fig. 14b). A standard curve of DEA NONOate was made using DEA NONOate solutions prepared in 10 mM of NaOH (Supplementary Fig. 14c).

### O_2_-dependence of the non-enzymatic triazole formation

For the anaerobic reactions, mixtures were prepared under N_2_ atmosphere in an anaerobic glovebox maintained at <0.5 ppm O_2_. Buffers and deionized water were thoroughly bubbled with Argon and brought into the glovebox and allowed for equilibrate for 48 h before use. Lyophilized **6** and solid powder of DEA NONOate were used for preparing stock solutions in the glovebox. A 200 μL reaction mixture consisting of **6** (75 μM) and DEA NONOate (1 mM) were prepared in a 1.5 mL Eppendorf tube (with the cap open) and allowed for equilibrate for 12 h before being transferred to an autosampler vial and brought out of the glovebox for UPLC-MS analysis using a Agilent Poroshell 120 EC-C18 column (2.7 μm, 2.1 mm ID × 100 mm) with isocratic elution (2% acetonitrile in water). Aerobic reaction mixtures were similarly prepared and left for 12 h on bench and used as positive control. The reaction of PtnB + **6** + NONOate was performed similarly as described above, except that PtnB (10 μM in phosphate buffer) was first treated with sodium dithionite (10 mM) in the glovebox and followed by extensive dialysis before the addition of **6** and NONOate.

### Chemical synthesis of **3**, **4**, and **8**

Small-scale chemical synthesis of **3**, **4**, and **8** were performed as follows (Supplementary Fig. 13b): to a 5 mL microwave reaction vial with a stir bar was added 1-*O-*acetyl-2,3,5-tri-*O*-benzoyl-*β*-D-ribofuranose (150 mg, 0.30 mmol), 8-azagunine (**1**) (107 mg, 0.45 mmol), 3 mL of acetonitrile, *N*,*O*-bis(trimethylsilyl)acetamide (0.328 mL, 1.34 mmol), and trimethylsilyl triflate (0.161 mL, 0.90 mmol). The vial was sealed with a mechanically crimped septum cap and heated in a microwave at 130 °C for 5 min. The contents were then transferred to a 40 mL glass vial and rinsed with 2 mL of acetonitrile. To this was added 1.33 mL of 1 M solution of triethanolamine (1.33 mmol) in acetonitrile with 2% water, and followed by shaking at room temperature for 30 min. The volatiles were removed by evaporation, and the residue was dissolved in 4 mL of 1:1 MeOH/CH_2_Cl_2_ and subjected to automated MPLC through a 12 g column of silica gel eluting at 30 ml min^−1^ with a gradient of 0–15% MeOH/CH_2_Cl_2_ over 30 min to provide 123 mg of **9** as amorphous solid (Supplementary Fig. 13b).

Fifty milligrams of **9** was dissolved in 5 mL methanol, and followed by the dropwise addition of 100 μL sodium methylate (5 M) at room temperature and stirred for 2 h. The mixture then was vacuum dried and subjected to HPLC separation to give 21 mg of **3** as white solid. Seven milligrams of proton-sponge and 10 mg of **3** were dissolved in 270 μl dry trimethyl phosphate and cooled to −15 °C under nitrogen atmosphere. After that, 5 μL POCl_3_ was added and the resulting mixture was stirred at −10 °C for 2 h. Fifty microliters of tributylamine and 158 mg tributylammonium pyrophosphate in acetonitrile solution were then added. Over 30 min, the reaction was allowed to warm slowly to 0 °C and was then quenched by addition of 600 μL aqueous triethylammonium bicarbonate (0.5 M, pH 7.5). The mixture was diluted with H_2_O and fractionized by a DEAE Sephadex column (GE Healthcare) and reverse-phase HPLC to give pure **8** and **4** (Supplementary Fig. 13c–f).

### Reporting summary

Further information on research design is available in the Nature Research Reporting Summary linked to this article.

## Supplementary information

Supplementary Information

Reporting Summary

## Data Availability

The data underlying the findings of this study are available in this article, Supplementary Information and Source data files (the source data underlying Figs. 3a, 4b, c and Supplementary Figs 3a, 3b, 6, 7, 10–12, 14, 15, 20 and 21b are provided as a Source Data file). The nucleotide sequence reported here have been deposited into GenBank (MN707952).

## Source data

Source Data
