# Supplementary material for: Nitric oxide as a source for bacterial triazole biosynthesis
Source: Nat Commun. 2020 Mar 31;11:1614. doi: 10.1038/s41467-020-15420-8 (PMC7109123; doi:10.1038/s41467-020-15420-8)
Supplement: Supplementary file 1 — Supplementary Information [file 41467_2020_15420_MOESM1_ESM.pdf]

Supplementary Information

Nitric Oxide as a Source for Bacterial Triazole Biosynthesis

Zhao *et al.*

Supplementary Table 1. The biosynthetic gene cluster of 8-azaguanine (pathocidin)

Gene	Size (aa)	(Putative) function
ptnA	206	GCH I-like protein
ptnB	111	Hypothetical protein
ptnC	167	Nucleosidase
ptnD	236	A didomain protein consisting of pyrophosphatase domain and *N*-acetyltransferase
ptnE	408	Purine efflux pump
ptnF	390	Nitric oxide synthase

NCBI accession number: MN707952 or NZ_LIQY01000068.1(7148..11814)

Supplementary Table 2. Primers used in this study

Primer name	Sequence (5′→3′)	Description
ptnF-RTF	CGAGCGATGTGCGTCAGGAAGTG	RT-PCR analysis of *ptnF*
ptnF-RTR	GTCTGTACTGGCAGTCGCTGATC	
ptnF-KOF	GGTAGCGCTGCTCGGTGTCGTAGTAGCGGTGGAACACGGATTCCG GGGATCCGTCGACC	In-frame deletion of *ptnF* in 29H7
ptnF-KOR	AGGTGGACAGCGCCGACCAGATCTCTCGGGAAATCGTCCGTGT AGGCTGGAGCTGCTTC	
ptnF-NdeI-F	AGCAGCCATATGAGTCCGGAGGAGGAGCGCTG	Cloning and expression of *ptnF,* confirmation of genotype of 29H7(Δ *ptnF*)
ptnF-XhoI-R	AGCAGCCTCGAGAGCAGGGAGGCCAAGGCTGGTTC	
ptnD-KOF	GATGCTGGCCCACGACGGTGACAACTGGCACATCCTGCTATTCCG GGGATCCGTC	In-frame deletion of *ptnD* in 29H7
ptnD-KOR	ACAGCCCACGAGGAAGCTCCCCAGTGCCGCTACGTGCGTCTGTAGG CTGGAGCTGCTTC	
ptnD-NdeI-F	AGCAGCCATATGCTGGCCCACGACGGTGACAAC	Confirmation of genotype of 29H7(Δ *ptnD*)
ptnD-XhoI-R	AGCAGCCTCGAGGTGGTGTGAGCGGTGTGGTCATGG	
ptnC-KOF	CATGGAGAAATGGCCGGATATCCTCATCGCCGTGGTCGGATTCCG GGGATCCGTCGACC	In-frame deletion of *ptnC* in 29H7
ptnC-KOR	GTGTGCTCGCCCACTGCCCATACGGTCTTGCCGGCGTCGATGT AGGCTGGAGCTGCTTC	
ptnC-NdeI-F	AGCAGCCATATGGAGAAATGGCCGGATATCCTC	Cloning and expression of *ptnC,* confirmation of genotype of 29H7(Δ *ptnC*)
ptnC-XhoI-R	AGCAGCCTCGAGATCACCAGGTCGACGGTCAGGATG	
ptnA-KOF	CACCATGGCTGAATCCCAGCAACCTCATTTACCGCTTCCATTCCGGGGATCCGTCGACC	Inactivation of *ptnA* in 29H7
ptnA-KOR	GTCTTCGTCGGCCACGGACGCCGACGGTGCTCCTCGGTCCATATGTCCGCCTCCTTTGGTC	
ptnA-YZF	CAAACGATCGTTCCGTGCAGACT	Confirmation of genotype of 29H7(Δ *ptnA*)
ptnA-YZR	AGAACAGGTGTGCAGTGCCTGTC	
ptnA-NdeI-F	AGCAGCCATATGGCTGAATCCCAGCAACCTC	Cloning and expression of *ptnA*
ptnA-XhoI-R	AGCAGCCTCGAGTCTCCCCAGGAAGGCCGGTTTC	
ptnD-NdeI-F	GCGGACATATGACTTTCGAAGTCG	Cloning of *txtD* (synthetic gene)
ptnD-XbaI-R	AGCAGCTCTAGATCACTGATGAGGGTAAAAGTTGGG	
ptnF-NdeI-F	AGCAGCCATATGAGTCCGGAGGAGGAGCGCTG	Construction of pYLD-*ptnF*

ptnF-XbaI-R	AGCAGCTCTAGAAGCAGGGAGGCCAAGGCTGGTTC	
ptnA-XbaI-F	AGCAGCTCTAGACACCACACGAACACCTGGCGTGT	Construction of pYLD-*ptnF-ptnABCDE*
ptnE-XbaI-R	AGCAGCTCTAGAGACTCACGGGGTTCGCCTGTGC	
ptnE-NdeI-F	AGCAGCCATATGACCACACCGCTCACACCACAC	Construction of pYLD-*ptnEF*
ptnF-XbaI-R	AGCAGCTCTAGAAGCAGGGAGGCCAAGGCTGGTTC	
ptnA-XbaI-F	AGCAGCTCTAGACACCACACGAACACCTGGCGTGT	Construction of pYLD-*ptnEF-ptnAB,* pYLD-*ptnF-patAB or ptnA*(H92A)B*
ptnB-XbaI-F	AGCAGCTCTAGACAGTTGCGTCCCTTGCCGACCAC	
ptnA-XbaI-F	AGCAGCTCTAGACACCACACGAACACCTGGCGTGT	Construction of pYLD-*ptnEF-ptnA*
ptnA-XbaI-R	AGCAGCTCTAGAGTGCTCCTCGGTCCATTCGGCTC	
*ptnA*p-OEPCR-P	CACTGCGCAGGACCATGGCGATTTGATCGACTGGGCATACAACTCCG	Construction of pYLD-*ptnEF-ptnB*
ptnB-OEPCR-P	CGGAGTTGTATGCCCAGTCGATCAAATCGCCATGGTCCTGCGCAGTG	
ptnA-XbaI-F	AGCAGCTCTAGACACCACACGAACACCTGGCGTGT	
ptnB-XbaI-F	AGCAGCTCTAGACAGTTGCGTCCCTTGCCGACCAC	
Ecoli-*RibA*-NdeI-F	AGCAGCCATATGCAGCTTAAACGTGTGGCAG	Expression of *E.coli* GCH II RibA
Ecoli-*RibA*-XhoI-R	AGCAGCCTCGAGTTATTTGTTCAGCAAATGGCCCAT	

Supplementary Table 3. Potential enzymes involved in nitrite/nitrate metabolism in *S. albus* J1074 and *S. albus* subsp. *pathocidicus* ATCC 14510.

Putative function	Locus_tag	Genbank ID
S. albus J1074		
Ferredoxin-nitrite reductase	XNR_0710	AGI87112
Nitrate reductase alpha chain	XNR_0412	AGI86814
Nitrate reductase beta chain	XNR_0413	AGI86815
Nitrate reductase delta chain	XNR_0414	AGI86816
Nitrate reductase gamma chain	XNR_0415	AGI86817
S. albus subsp. *pathocidicus* ATCC 14510		
Nitrite reductase large subunit		WP_055470768
Nitrite reductase small subunit		WP_055470767
Nitrite/sulphite reductase		WP_055472164
Nitrate reductase gamma chain		WP_150512515
Cytochrome C nitrite reductase		WP_055473410

Supplementary Fig. 1.

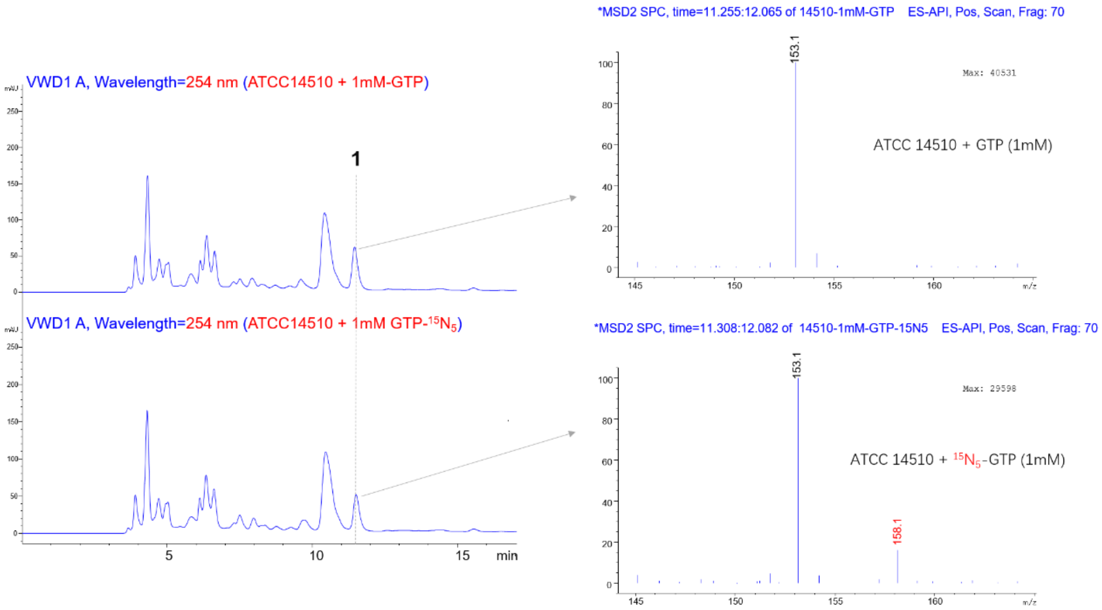

Supplementary Fig. 1. LC-MS analysis of the culture supernatant of *Streptomyces albus subsp. pathocidicus* ATCC 14510 fed with GTP or $^{15}N_5$-GTP at final concentrations of 1 mM at 24 h. Left panel, HPLC traces detected at the wavelength of 254 nm. Right panel, extracted MS data for 8-azaguanine (**1**) (*m*/*z* 153, $[M+H]^+$) and $^{15}N_5$-**1** (*m*/*z* 158, $[M+H]^+$).

Supplementary Fig. 2.

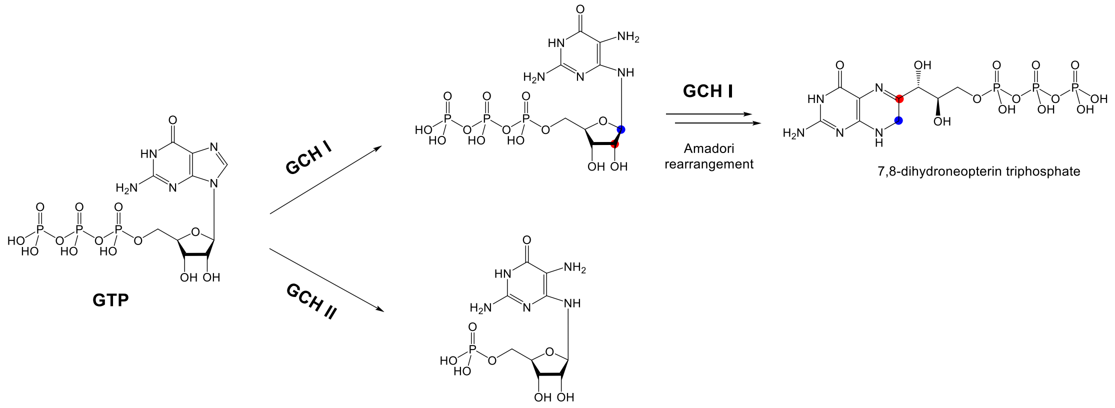

Supplementary Fig. 2. Reactions catalyzed by GCH I and II. GCH I is part of the folate and biopterin biosynthetic pathways and is responsible for the multistep conversion of GTP to 7,8-dihydroneopterin triphosphate. GCH II is involved in riboflavin metabolism, and catalyzes the hydrolytic opening of the imidazole ring of GTP, with concomitant removal of a pyrophosphate moiety[1]. Although GCH I and II share no sequence homology, they both rely on an essential zinc ion for hydrolysis of the imidazole ring of GTP and release C8 as formic acid.

Supplementary Fig. 3.

(a) **(b)**

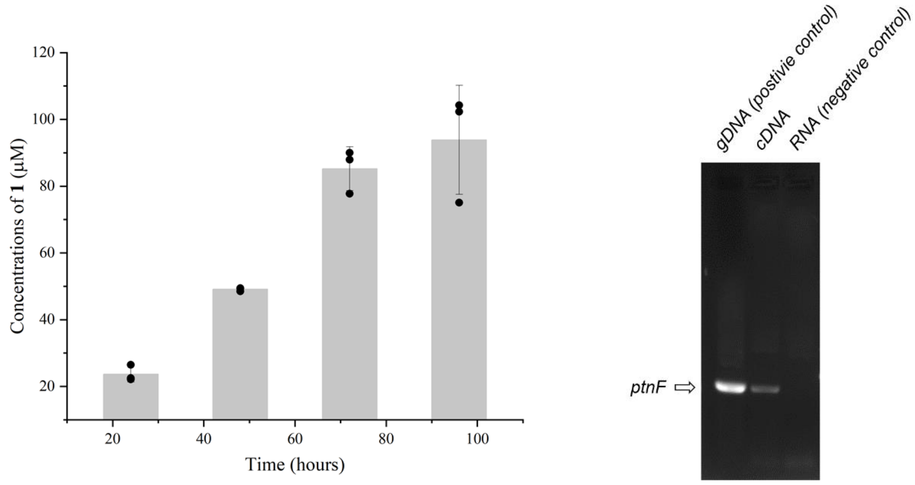

Supplementary Fig. 3. Production of **1** by *Streptomyces albus subsp. pathocidicus* ATCC 14510. (**a**) Time-course curve of 8-azaguanine production by *Streptomyces albus subsp. pathocidicus* ATCC 14510. Values are means of three independent experiments ± SD. (**b**) RT-PCR analysis of the *ptnF* gene using template cDNA from the mycelium of *Streptomyces albus subsp. pathocidicus* ATCC 14510 harvested at 36 h. The experiment was repeated twice independently and the representative data was shown. Source data are provided as a Source Data file.

Supplementary Fig. 4.

(a)

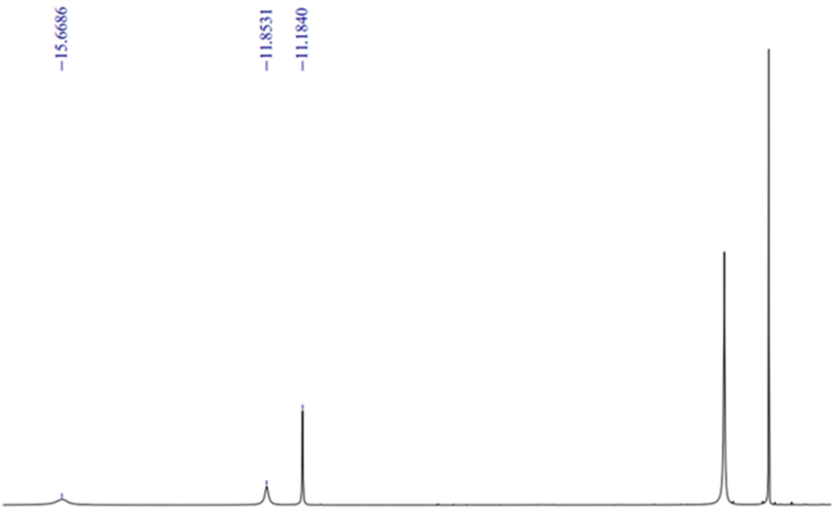

(b)

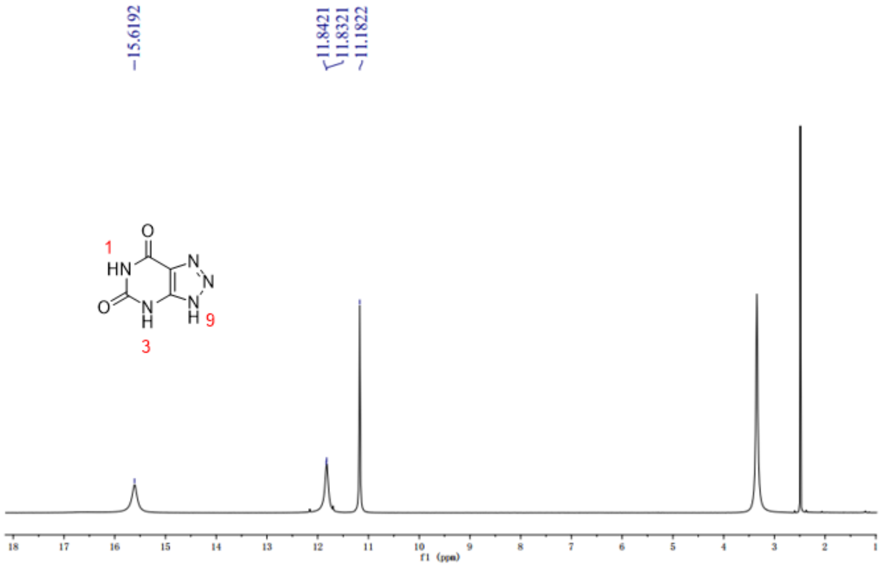

Supplementary Fig. 4. ^{1}H NMR spectra of compound **2** (**a**) and authentic 8-azaxanthine (**b**). Compound **2**: ESIMS *m/z* 152.10 [M-H]$^-$ (calcd for $C_4H_3N_5O_2$ 152.10); ^{1}H NMR (600 MHz, DMSO-d_6) 11.1 (s, NH-3), 11.8 (s, NH-1), 15.7 (s, NH-7); 8-Azaxanthine: ESIMS *m/z* 152.10 [M-H]$^-$ (calcd for $C_4H_3N_5O_2$ 152.10); ^{1}H NMR (600 MHz, DMSO-d_6) 11.1 (s, NH-3), 11.8 (s, NH-1), 15.6 (s, NH-9).

Supplementary Fig. 5.

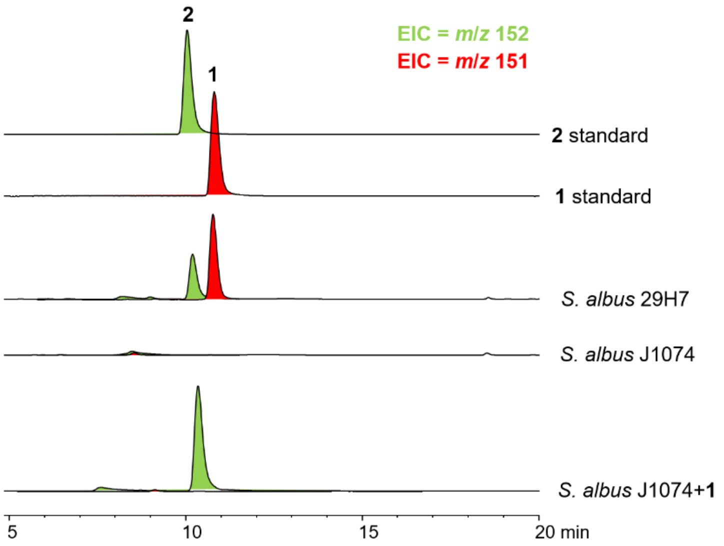

Supplementary Fig. 5. LC-MS analysis revealing that 8-azaxanthine (**2**) (*m/z* 152, $[M-H]^-$) is a host-modified metabolite of 8-azaguanine (**1**) (*m/z* 151, $[M-H]^-$). Note: compound **1** was fed to the culture broth of *S. albus* J1074 at 24h, with a final concentration of 0.1 mM. LC-MS analysis was performed with the culture supernatant from 96 h.

Supplementary Fig. 6.

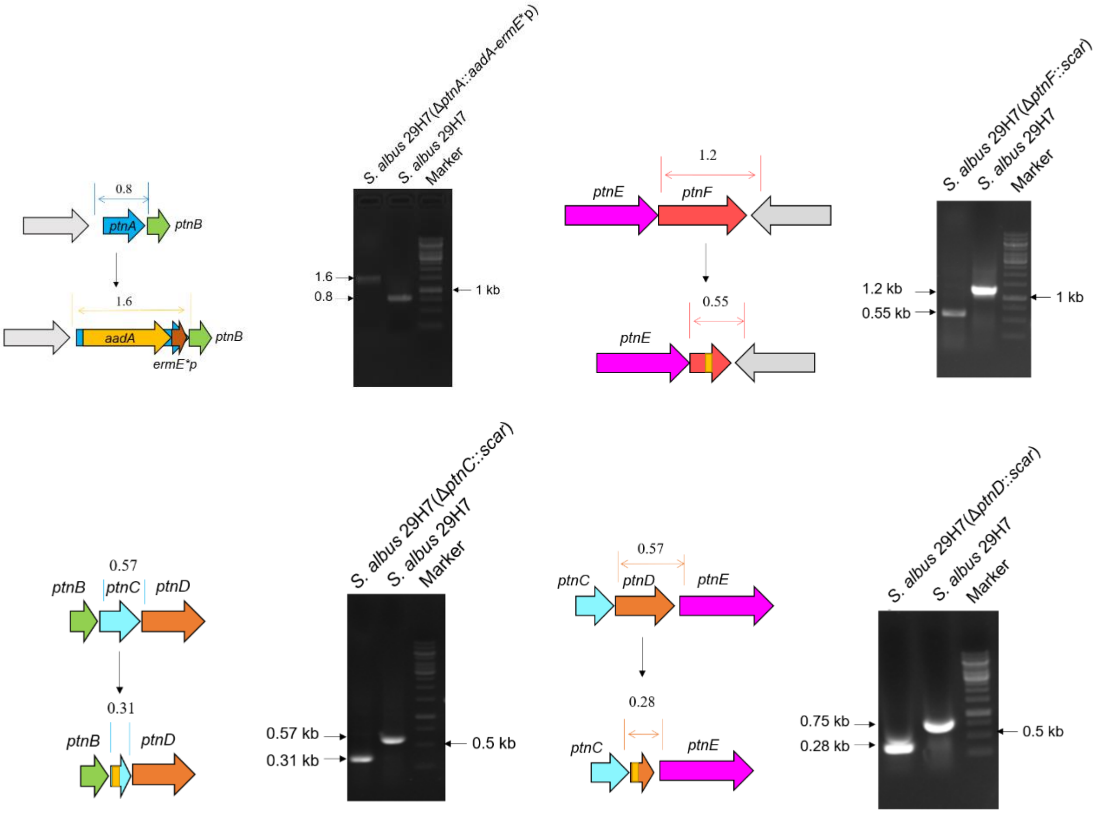

Supplementary Fig. 6. Construction of the gene-inactivation mutants and confirmation of their genotype by PCR using genomic DNA from the corresponding *S. albus* mutants as templates. The experiment was repeated four times independently and the representative data was shown. Source data are provided as a Source Data file.

Supplementary Fig. 7.

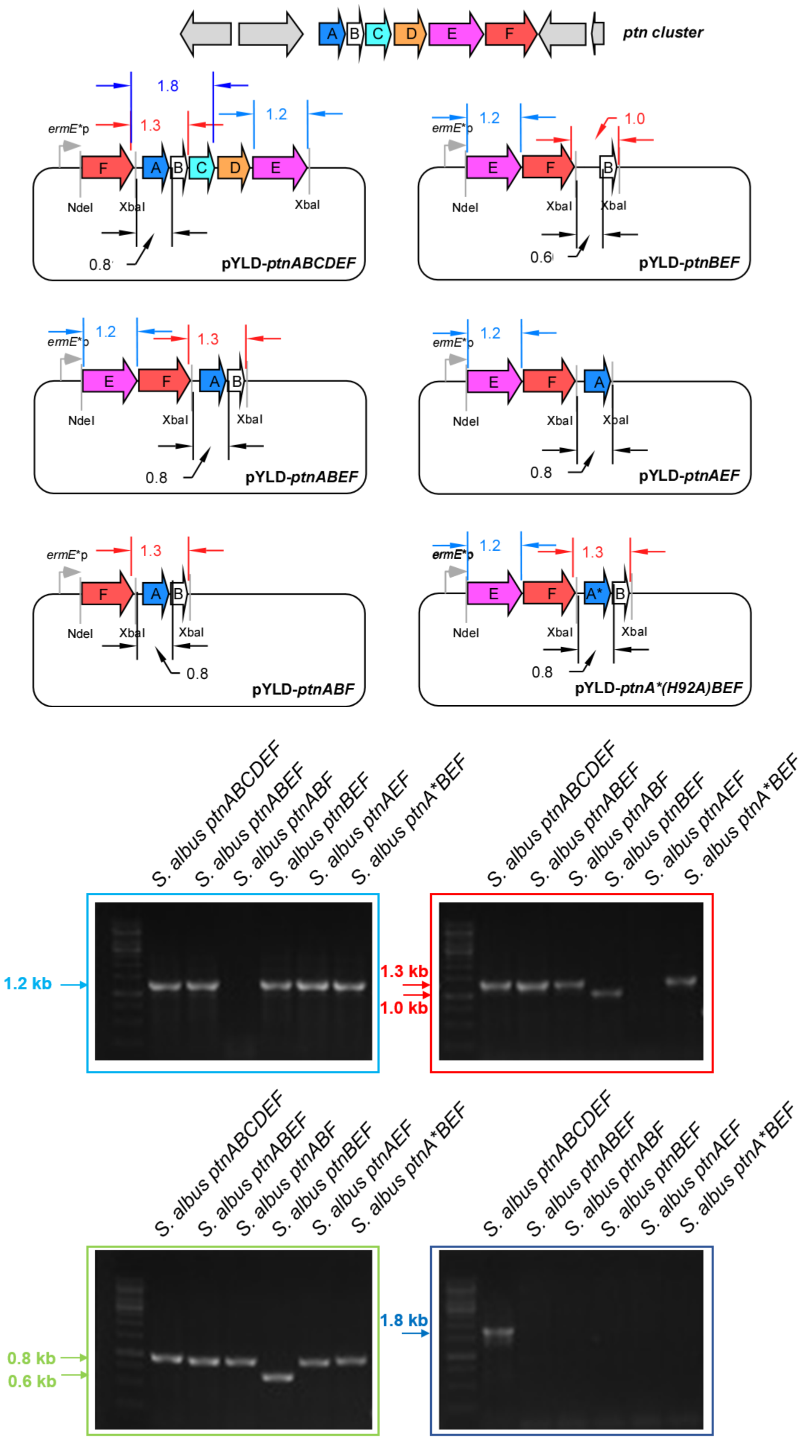

Supplementary Fig. 7. Construction of vectors for *in vivo* reconstitution and confirmation of the integration of vectors in *S. albus* genome. Six vectors were constructed as shown in the top panel. Four pairs of primers (listed in **Supplementary Table 2** and shown as different-colored arrows in the top panel) were used to amplify the corresponding region from engineered *S. albus* strains (bottom) for the confirmation of their genotype. The experiment was repeated four times independently and the representative data was shown. Source data are provided as a Source Data file.

Supplementary Fig. 8.

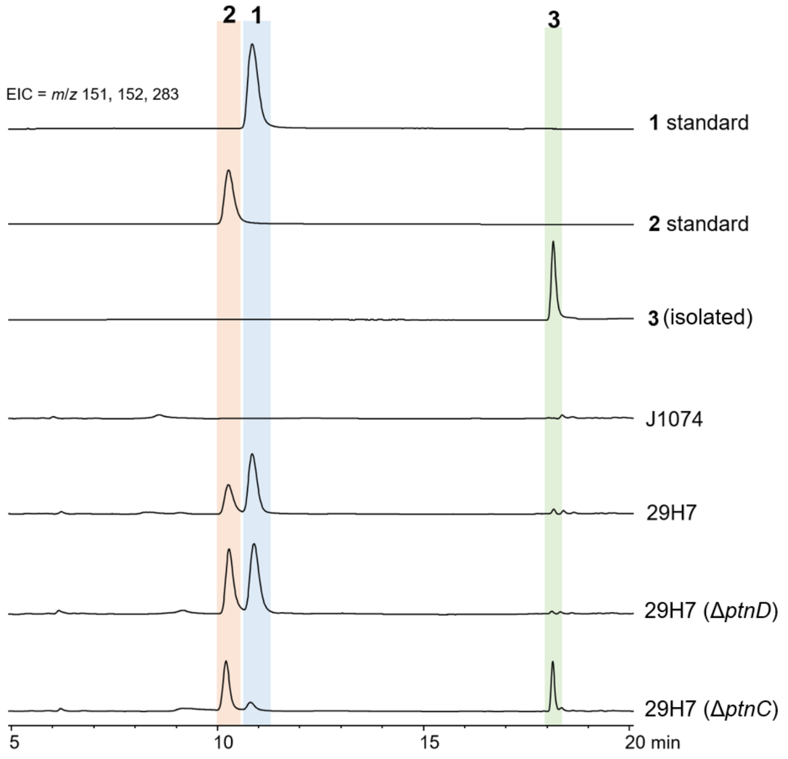

Supplementary Fig. 8. LC-MS analysis of strain *S. albus* 29H7(Δ*ptnC*) and *S. albus* 29H7(Δ*ptnD*) showing that compound **3** only accumulates in *S. albus* 29H7(Δ*ptnC*).

Supplementary Fig. 9.

(a)

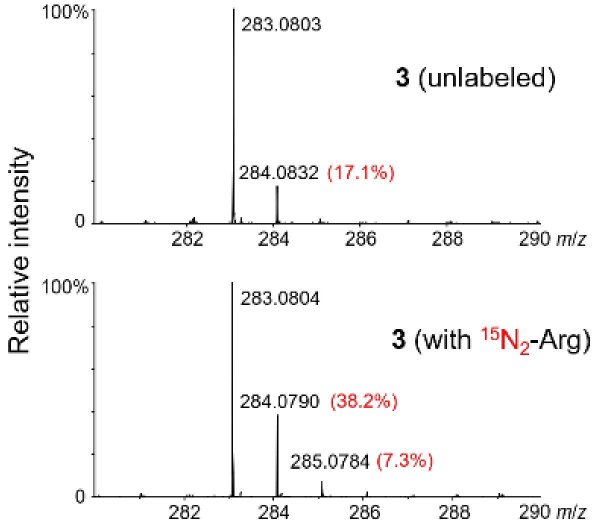

(b)

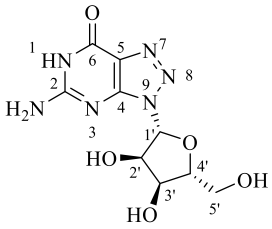

	Compound 3[a]				
**NO.**	δ_H	δ_C	HSQC	COSY	HMBC
**2**	_	158.6	-	-	-
**4**	_	154.5	-	-	-
**5**	_	127.3	-	-	-
**6**	_	160.8	-	-	-
**1′**	6.16 (1H, d, J = 4.2 Hz)	91.6	C-1′	H-2′	C-4, C-2′, C-3′, C-4′
**2′**	4.96 (1H, t, J = 4.7 Hz)	76.3	C-2′	H-1′, H-3′	C-1′, C-3′
**3′**	4.59 (1H, t, J = 5.1 Hz)	73.4	C-3′	H-3′, H-4′	C-1′, C-5′
**4′**	4.25 (1H, td, J = 5.1, 3.2 Hz)	88.1	C-4′	H-3′, H-5′	C-1′, C-3′
**5′ a,b**	3.75 (1H, dd, J = 12.7, 3.2 Hz)<br>3.86 (1H, dd, J = 12.7, 5.2 Hz)	64.3	C-5′,	H-4′	C-3′, C-4′

[a] ^{1}H (600 MHz) and ^{13}C (150 MHz) NMR Data in D_2O

(c)

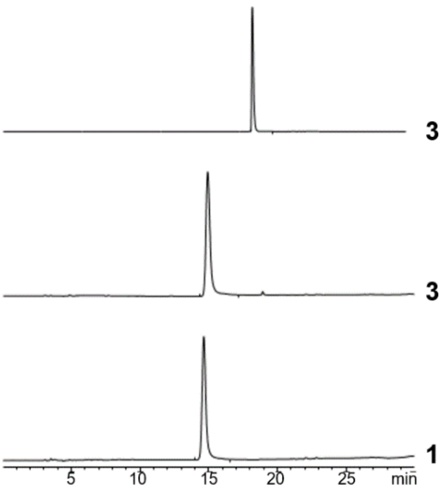
3
3 in 1N HCl at 98℃ for 1h
1
5
10
15
20
25
min

(d)

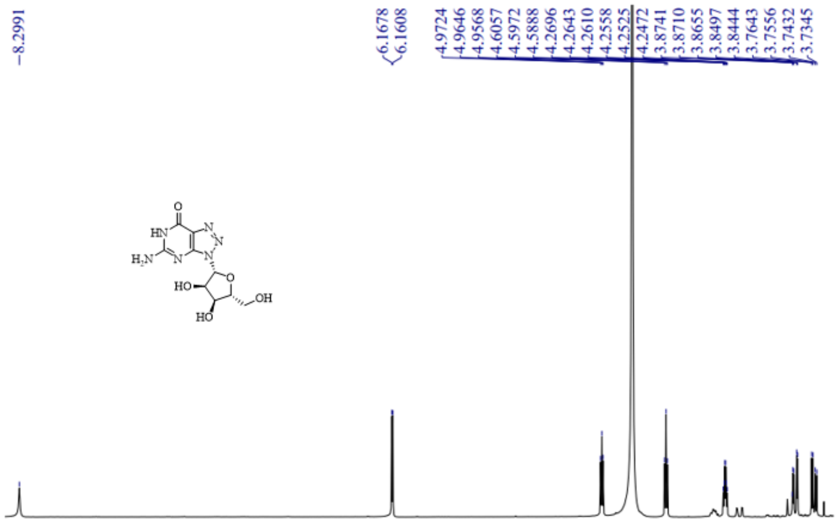
8.2991
6.1678
6.1608
4.9724
4.9646
4.9568
4.6057
4.5972
4.5888
4.2696
4.2643
4.2610
4.2558
4.2525
4.2472
3.8741
3.8710
3.8655
3.8497
3.8444
3.7643
3.7556
3.7432
3.7345

(e)

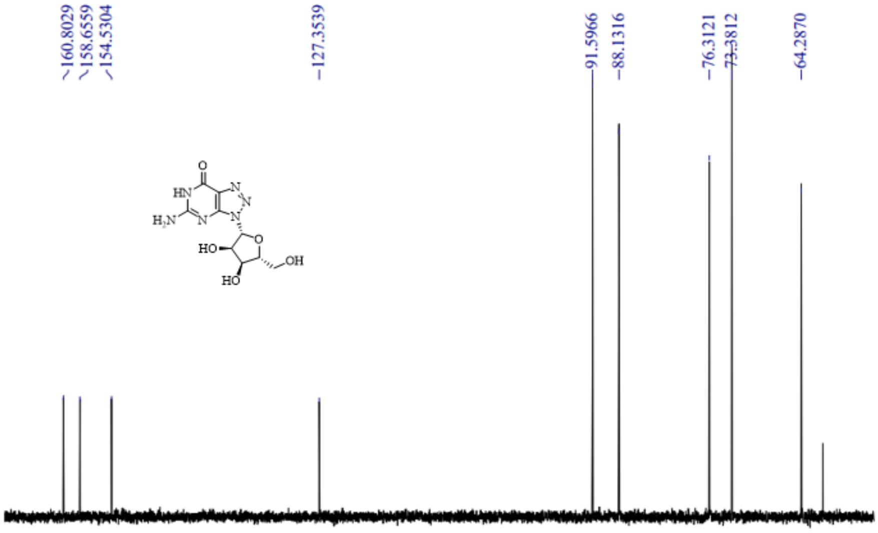
160.8029
158.6559
154.5304
127.3539
91.5966
88.1316
76.3121
73.3812
64.2870

(f)

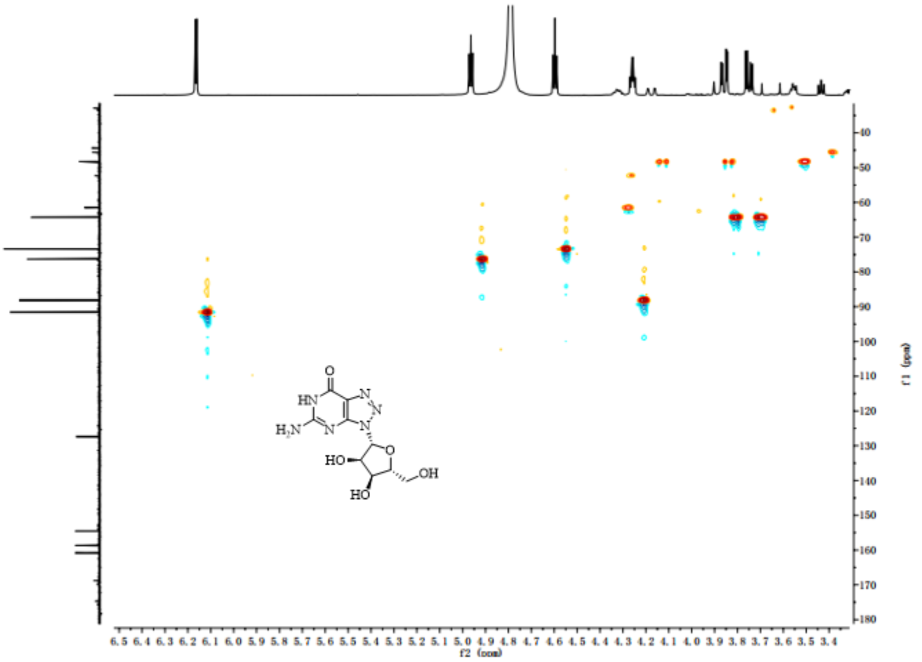

(g)

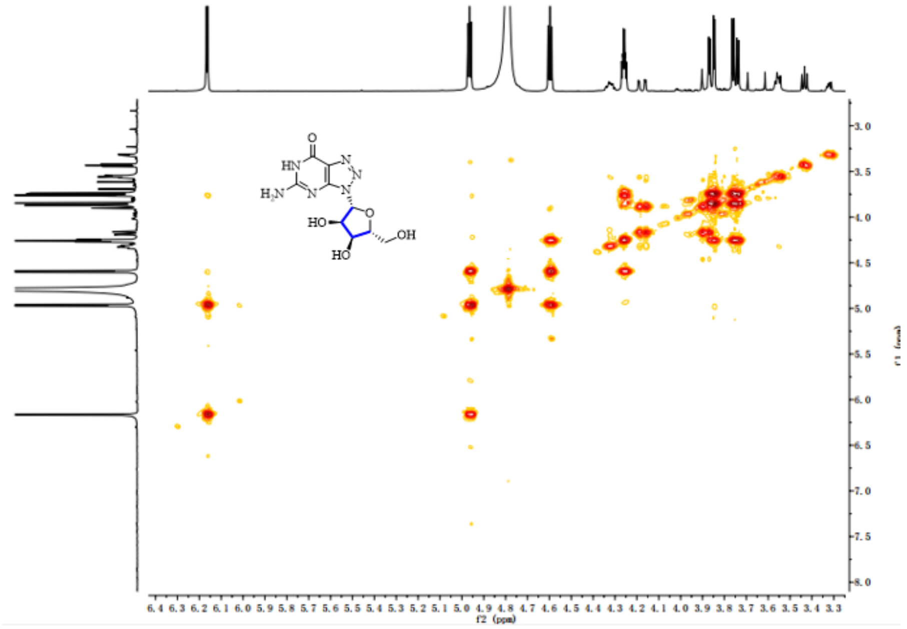

(h)

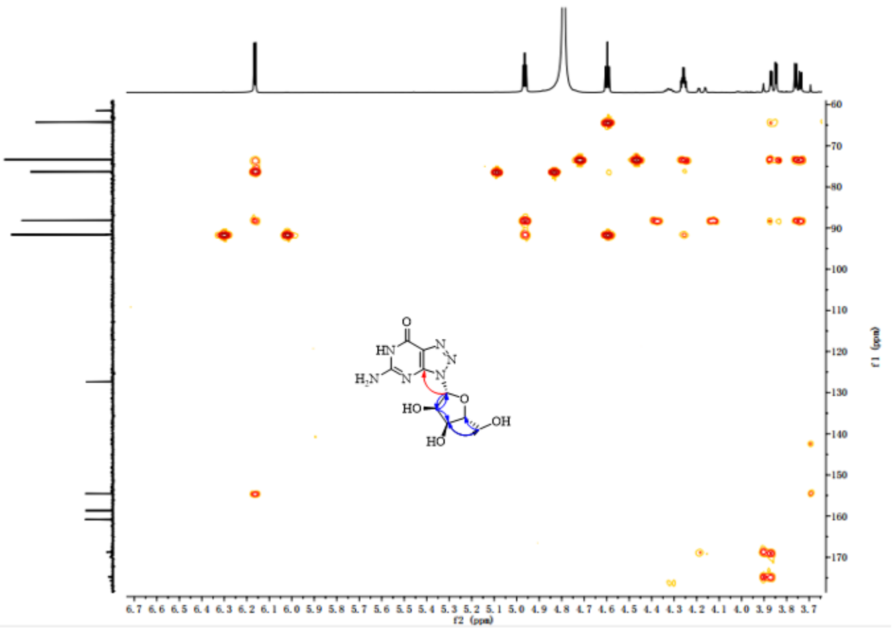

Supplementary Fig. 9. Characterization of compound **3**. (**a**) LC-HR-ESI-MS of compound **3**. (**b**) Summary of the NMR data for **3**. (**c**) Acidic hydrolysis of the *N*-glycosidic bond of **3** for the confirmation of structure elucidation. (**d**) ^{1}H NMR spectrum of compound **3** (600 MHz, D_2O). (**e**) ^{13}C NMR spectrum of compound **3** (150 MHz, D_2O). (**f**) HSQC spectrum of compound **3** in D_2O. (**g**) ^{1}H-^{1}H-COSY spectrum of compound **3** in D_2O. (**h**) HMBC spectrum of compound **3** in D_2O. Compound **3** HR-ESIMS *m/z* 283.0804 [M - H]$^-$ (calcd. for $C_9H_{12}N_6O_5$ 283.0869); ^{1}H NMR (600 MHz, D_2O) 6.16 (1H, d, J = 4.2 Hz, H-1′), 4.96 (1H, t, J = 4.7 Hz, H-2′), 4.59 (1H, t, J = 5.1 Hz, H-3′), 4.25 (1H, td, J = 5.1, 3.2 Hz, H-4′), 3.75 (1H, dd, J = 12.7, 3.2Hz, H-5′b), 3.86 (1H, dd, J = 12.7, 5.2 Hz, H-5′a); ^{13}C NMR (150 MHz, D_2O) 158.6 (s, C-2), 154.5 (s, C-4), 127.3 (s, C-5), 160.8 (s, C-6), 91.6 (s, C-1′), 76.3 (s, C-2′), 73.4 (s, C-3′), 88.1 (s, C-4′), 64.3 (s, C-5′).

Supplementary Fig. 10.

(**a**)

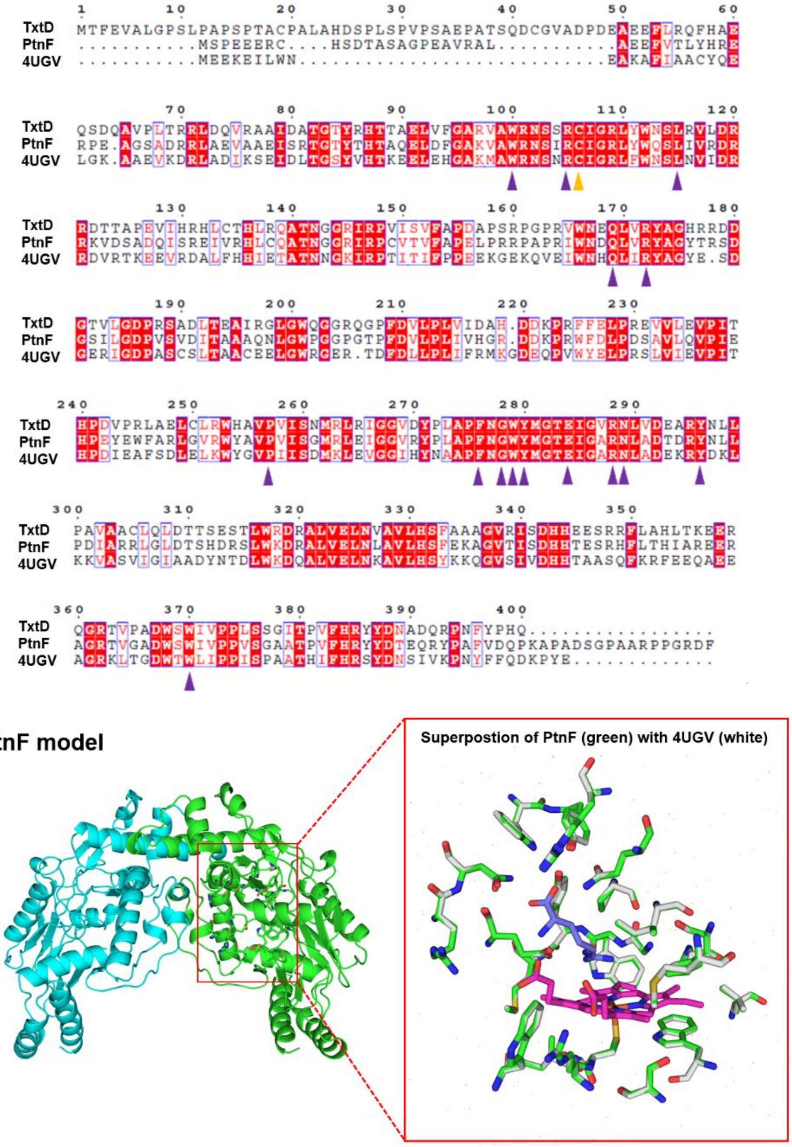

(**b**)

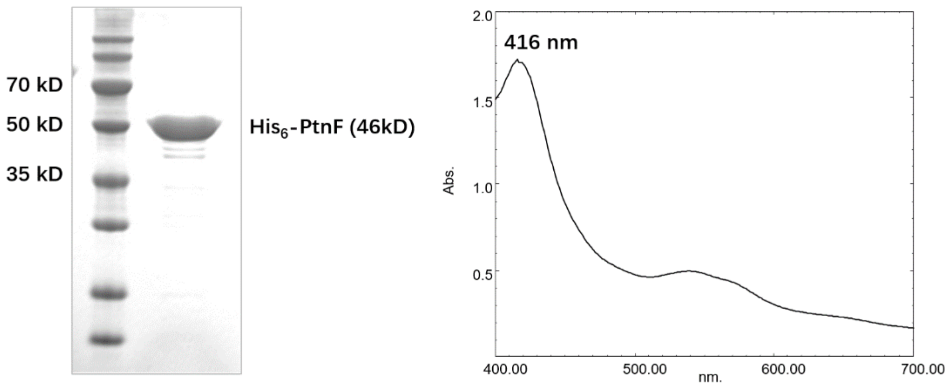

(**c**)

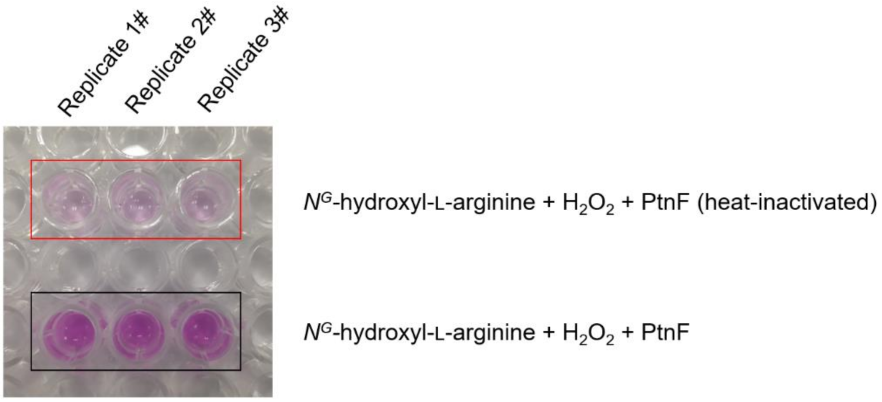

Supplementary Fig. 10. In vitro characterization of PtnF. (**a**) Sequence alignment of PtnF with characterized NOS TxtD (Genbank accession number: AAO53227) and *Bacillus subtilis* NOS (PDB: 4UGV). The figure was generated by ClustalX2[2], ESPript 3.0[3] and Pymol[4]. Conserved active site residues are indicated with purple triangles, heme-ligating cysteine residue is indicated with an orange triangle. The homology model of PtnF was generated by SWISS-MODEL. The heme cofactor is indicated in purple, and the substrate arginine is indicated in blue. (**b**) SDS-PAGE and UV-Vis spectrum of isolated His_6-PtnF. The experiment was repeated twice independently and the representative data was shown. Source data are provided as a Source Data file. (**c**) Biochemical assay of PtnF. Nitrite production was quantified by using Griess Reagents as described in Methods.

Supplementary Fig. 11.

(**a**)

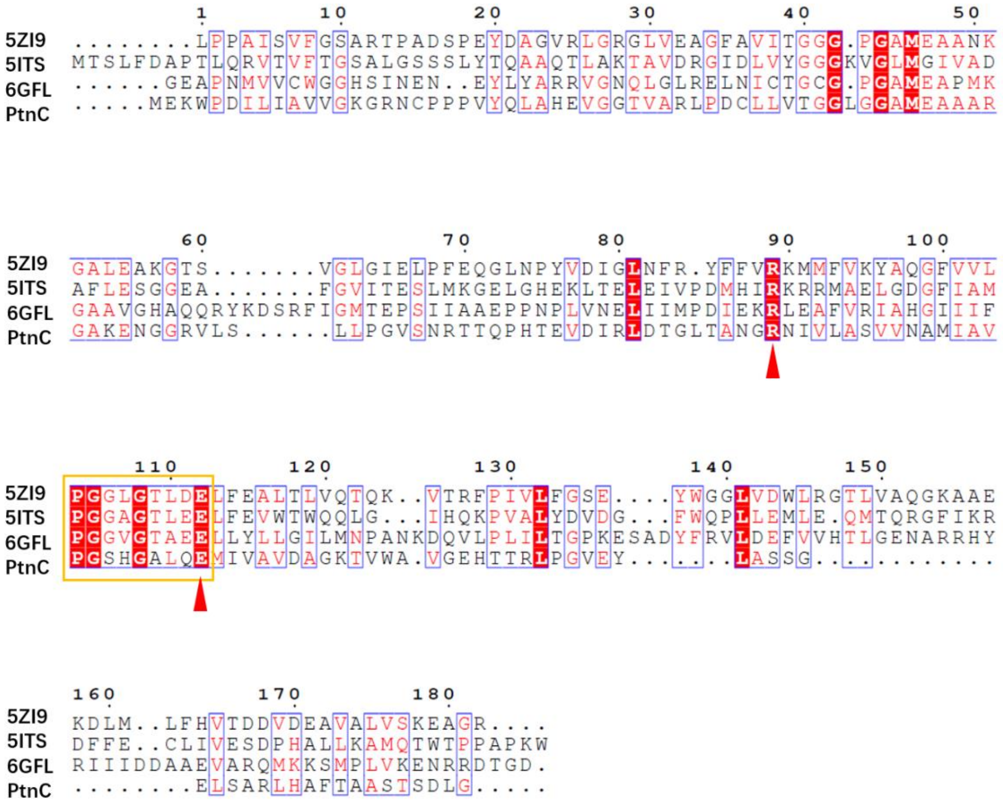

(**b**)

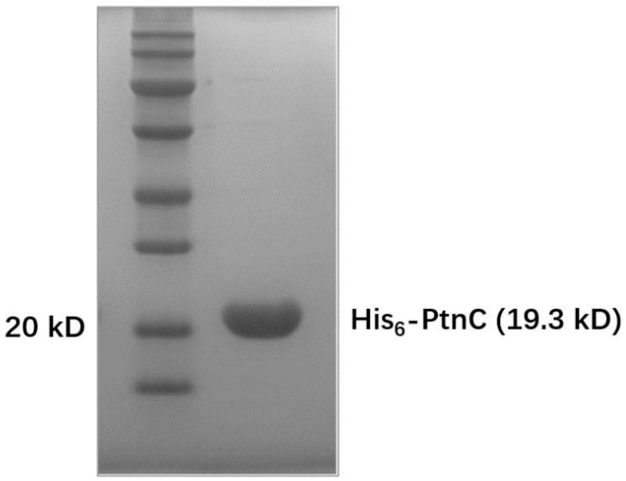

(**c**)

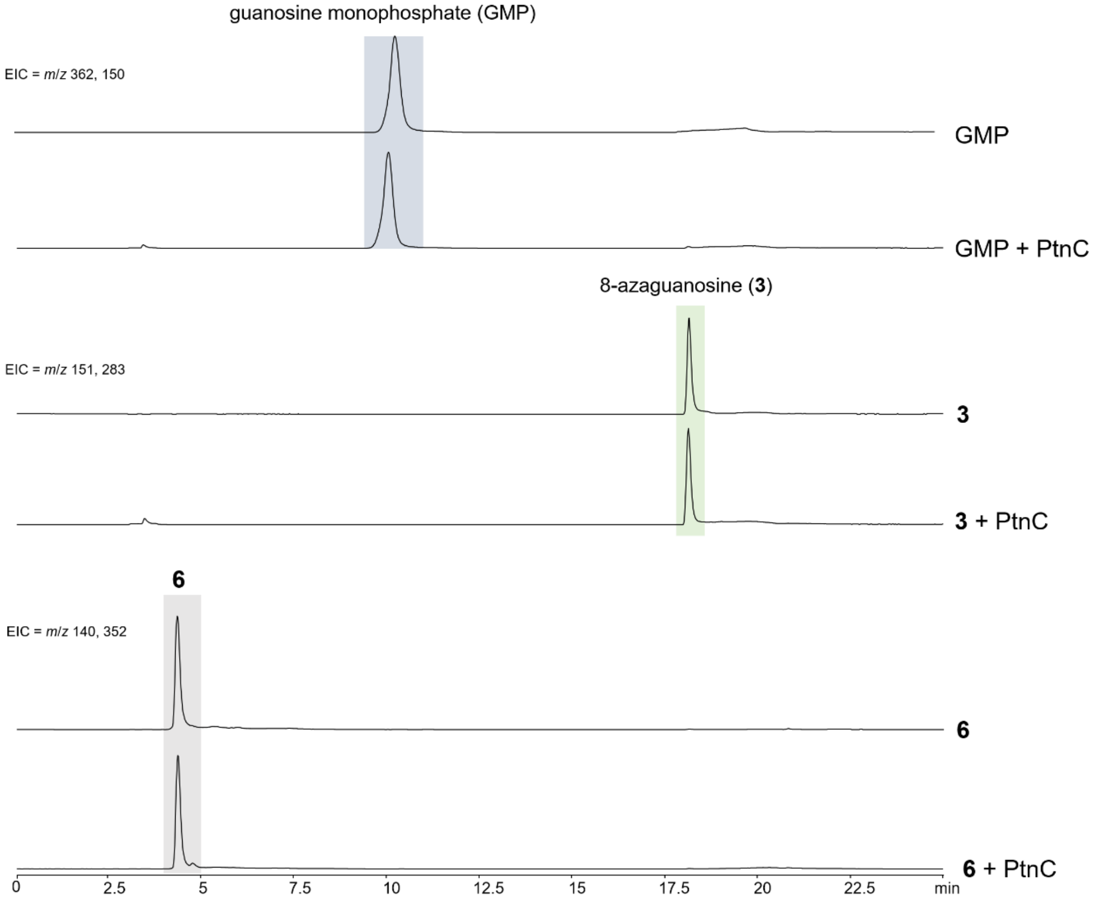

Supplementary Fig. 11. In vitro characterization of PtnC. (**a**) Sequence alignment of PtnC with characterized cytokinin riboside 5'-monophosphate phosphoribohydrolase from *Streptomyces coelicolor* M145 (PDB: 5ZI9) and *Corynebacterium glutamicum* ATCC 13032 (PDB: 5ITS), and the catalytic domain of *E. coli* pyrimidine/purine nucleotide 5'-monophosphate nucleosidase PpnN (PDB: 6GFL). Conserved active site residues involved in catalysis are indicated with red triangles, and putative substrate binding motif is boxed with an orange rectangle. (**b**) SDS-PAGE of isolated His_6-PtnC. The experiment was repeated twice independently and the representative data was shown. Source data are provided as a Source Data file. (**c**) Incubation of PtnC (20 μM) with GMP (0.1 mM), 8-azaguanosine (**3**) (0.1 mM), or 2,5-diamino-6-ribosylamino-4(3*H*)-pyrimidinone 5′-phosphate (**6**) (0.1 mM) gave no detectable product.

Supplementary Fig. 12.

(**a**)

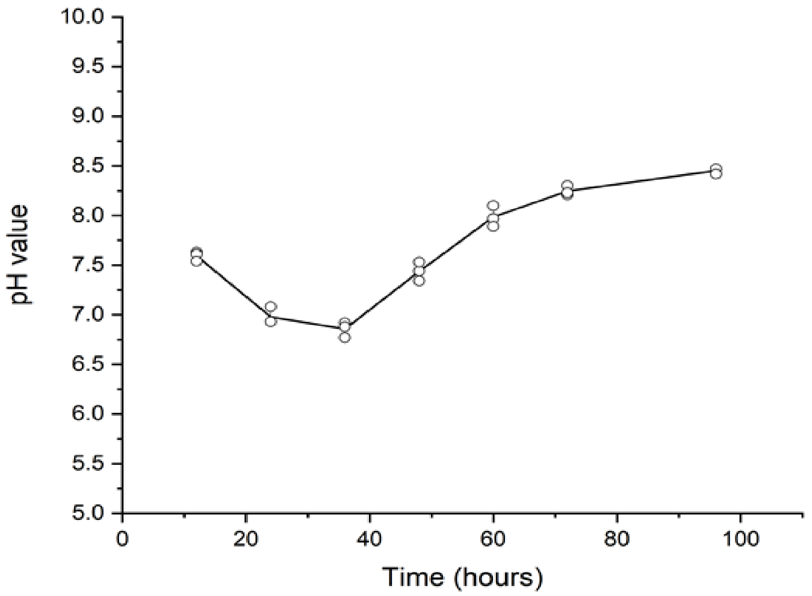

(**b**)

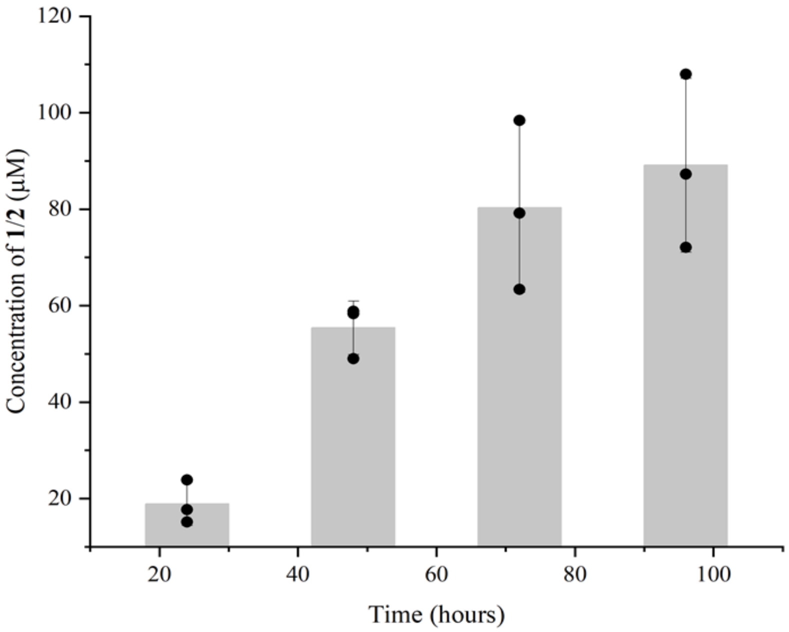

Supplementary Fig. 12. Time-course curve of pH values (**a**) and production of compound **1** and **2** (**b**) by *S. albus* 29H7. Values are means of three independent experiments ± SD. Source data are provided as a Source Data file

Supplementary Fig. 13.

(a)

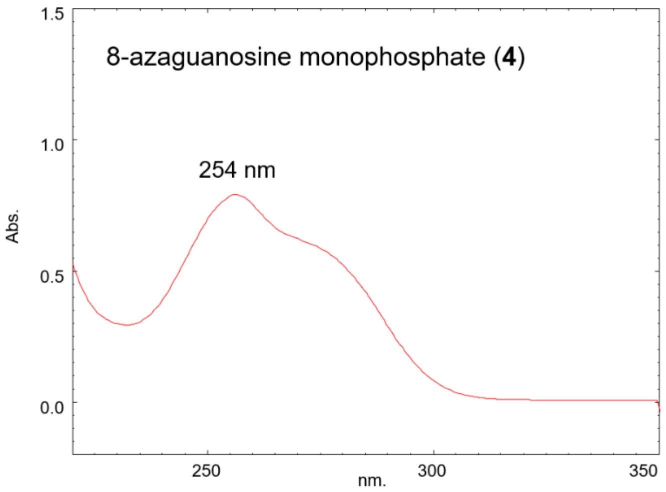

(b)

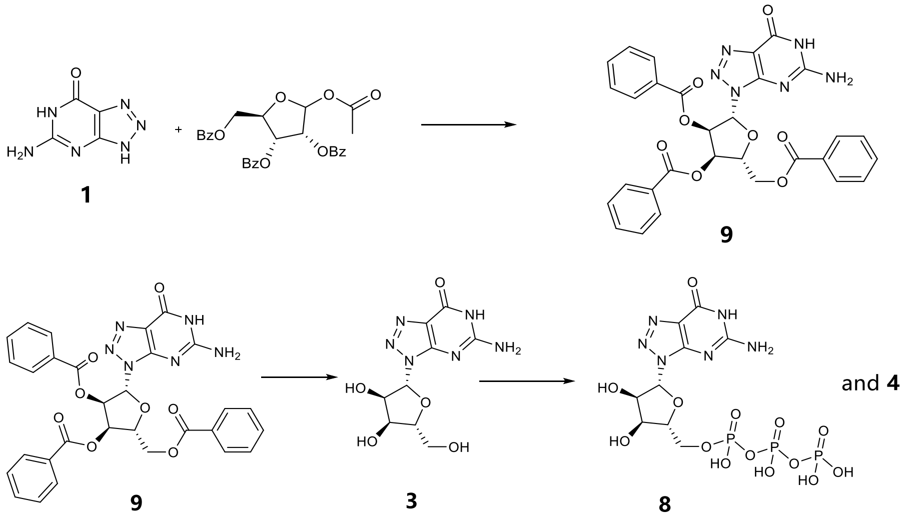

(c)

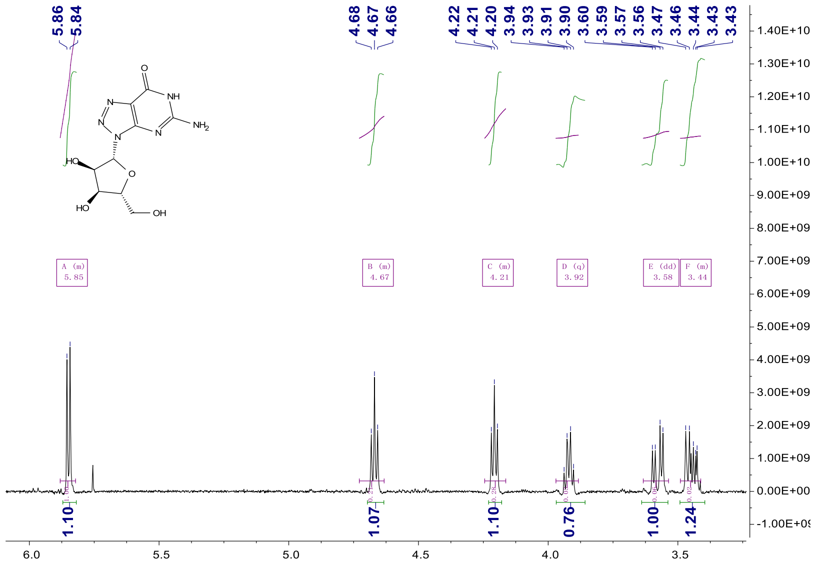

(d)

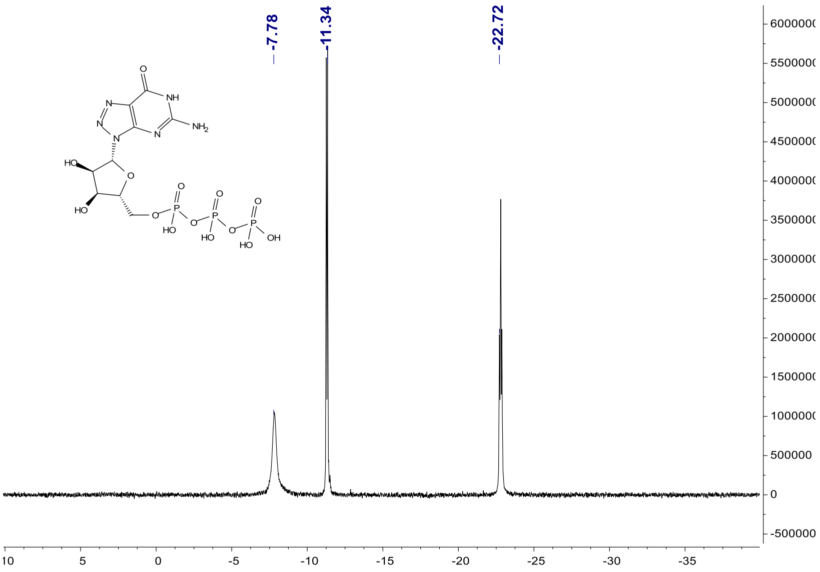

(e)

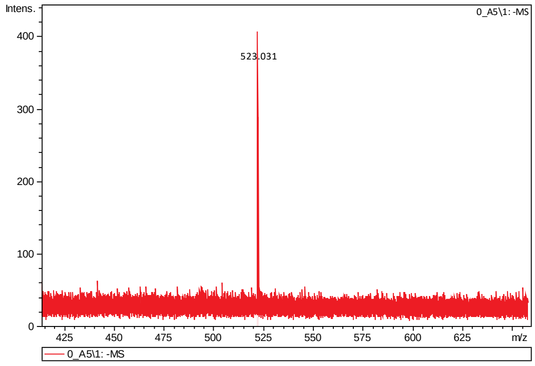

(f)

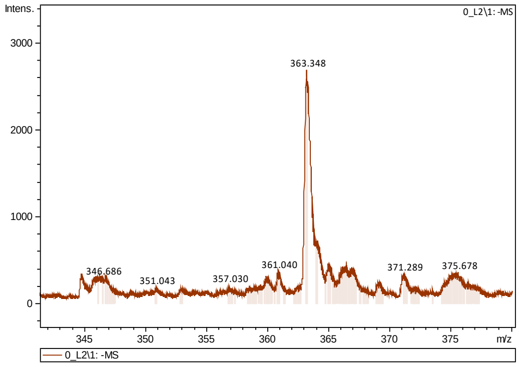

Supplementary Fig. 13. UV-vis spectrum of synthetic **4** and chemical synthesis of **4**, **3**, and 8-azaguanosine triphosphate (**8)** as analytic standards. (**a**) UV-vis spectrum of authentic **4** and **8**. (**b**) Synthetic routes to **3**, **4** and **8** (See Methods). (**c**) ^{1}H NMR spectrum of **3** in DMSO-d^6. (**d**) ^{31}P NMR spectrum of **8** in D_2O. (**e**) and (**f**) Mass analysis of synthetic **8** (**e**) and **4** (**f**) under negative mode. Compound **9**: ^{1}H NMR (400 MHz, Chloroform-*d*) δ 8.16 – 7.90 (m, 6H), 7.60 – 7.27 (m, 9H), 6.81 (s, 1H), 6.58 (s, 1H), 6.35 (s, 1H), 4.88 (d, J = 40.7 Hz, 3H). Compound **3**: ^{1}H NMR (400 MHz, DMSO-d_6) δ 5.88 – 5.82 (m, 1H), 4.73 – 4.63 (m, 1H), 4.24 – 4.16 (m, 1H), 3.92 (q, J = 4.6 Hz, 1H), 3.58 (dd, J = 11.8, 4.4 Hz, 1H), 3.49 – 3.41 (m, 1H). Compound **8**: ^{31}P NMR (243 MHz, D_2O) δ -7.78 (s), -11.30 (d, J = 19.6 Hz), -22.80 (t, J = 19.8 Hz); MS (MALDI-TOF, *m/z*) $[M-H]^-$ Calculated for $C_9H_{14}N_6O_{14}P_3$, 523.160; found, 523.031.

Supplementary Fig. 14.

(**a**)

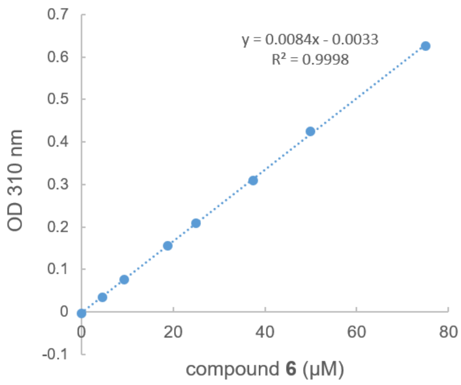

(**b**) (**c**)

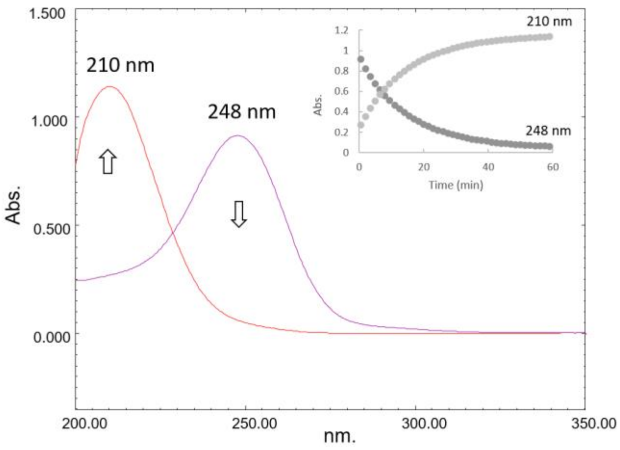

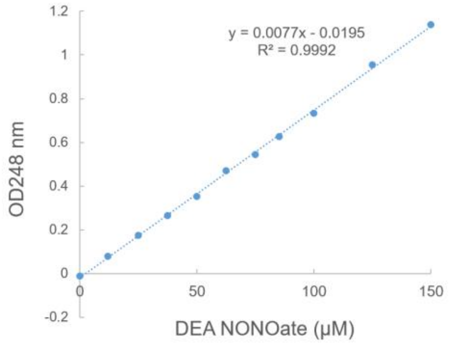

Supplementary Fig. 14. Standard curves of **6** and UV-Vis spectroscopic studies of the decomposition of DEA NONOate in pH 7.4 phosphate buffer at 25℃. **(a)** Standard curve of **6** prepared in pH 7.4 phosphate buffer. **(b)** Change of DEA NONOate (150 μM, purple line) absorption spectra upon its addition into pH 7.4 phosphate buffer at 25°C. The spectrum collected 1 h later is shown in red line. The absorbance at 248 and 210 nm were monitored with a 1.5-min time interval (as shown in the insert figure). (**c**) Standard curve of DEA NONOate prepared in 10 mM of NaOH solution. Source data are provided as a Source Data file.

Supplementary Fig. 15.

(a) **(b)**

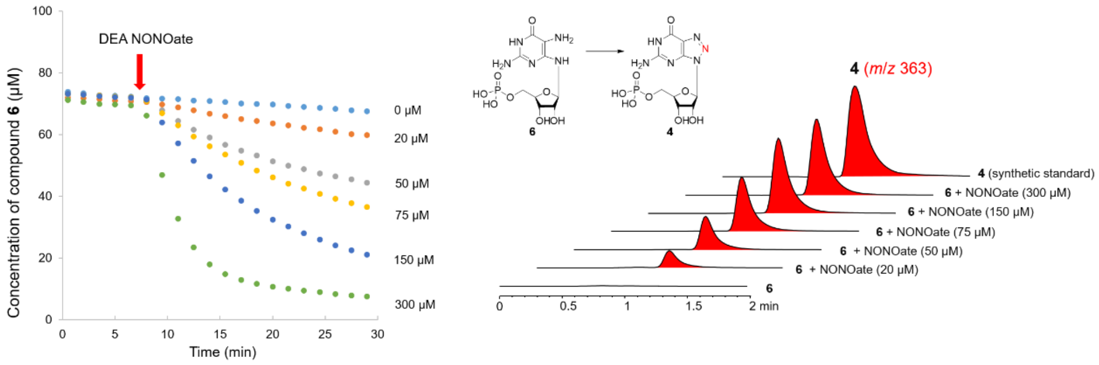

Supplementary Fig. 15. Conversion of **6** to **4** in the presence of difference concentrations of NO donor DEA NONOate. **(a)** Consumption of **6** (75 μM) over time upon the addition of DEA NONOate (at the final concentration of 0 μM, 20 μM, 50 μM, 75 μM, 150 μM, 300 μM). The red arrow indicates the time point when DEA NONOate was added. The compound **6** was quantified based on its absorbance at 310 nm (**Supplementary Fig. 14**). These reaction mixtures were monitored by UV-Vis spectroscopic analysis with a 1.5-min time interval. The rate of consumption of **6** increases as the DEA NONOate concentration increases. Note: compound **6** is not stable and decomposes over time, with a half-life of a few hours[5]. Source data are provided as a Source Data file. **(b)** UPLC-MS analysis (EIC = *m*/*z* 363) of the reaction mixtures from **(a)** after 1 h.

Supplementary Fig. 16.

(a)

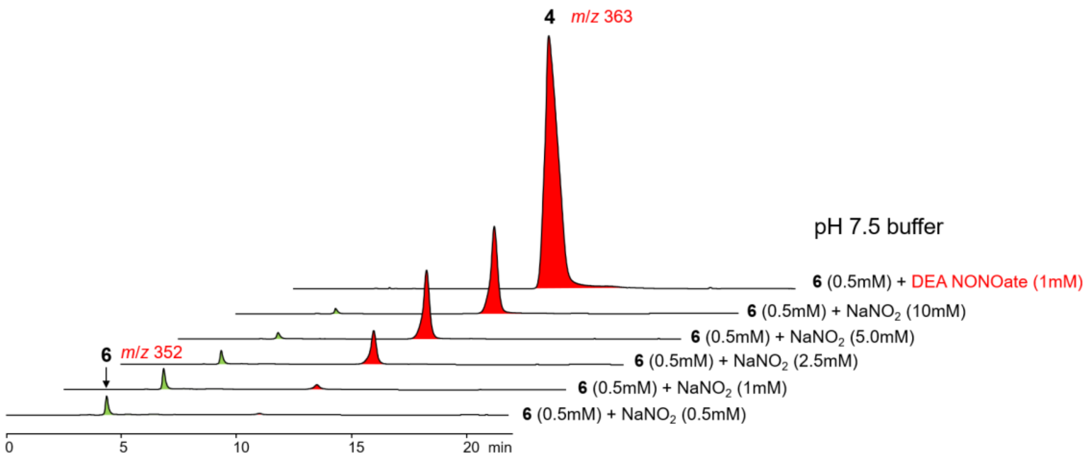

(b)

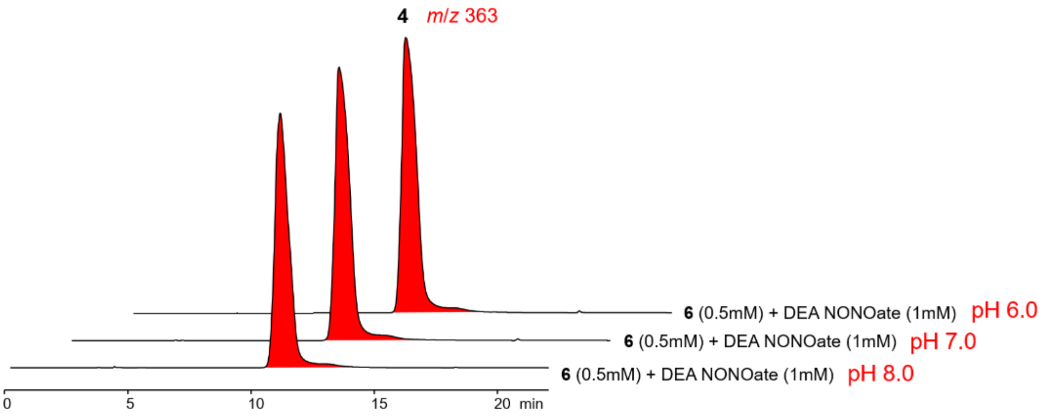

Supplementary Fig. 16. Investigation of the effects of nitrite concentration (**a**) and pH values (**b**) on the non-enzymatic triazole formation by LC-MS (EIC = *m*/*z* 352 and 363, $[M\text{-}H]^-$ of **4** and **6**, respectively). (**a**) Reaction of **6** (0.5 mM) with sodium nitrite (0.5 mM, 1 mM, 2.5 mM, 5 mM, 10 mM) in Tris buffer (pH 7.5). Only partial conversion of **6** to **4** could be achieved even at a very high concentration of sodium nitrite (10 mM), whereas full conversion of **6** to **4** was observed with 1 mM DEA NONOate. Note: the ionization efficiency of compound **6** is not as good as **4** under the negative mode. (**b**) Conversion of **6** to **4** in Tris buffers with different pH values (6.0, 7.0, and 8.0). All the reactions were incubated at 30°C for 1 hr before LC-MS analysis. Full conversion of **6** to **4** was observed (in the presence of 2 equivalents of DEA NONOate) in all the buffers we tested.

Supplementary Fig. 17.

(a)

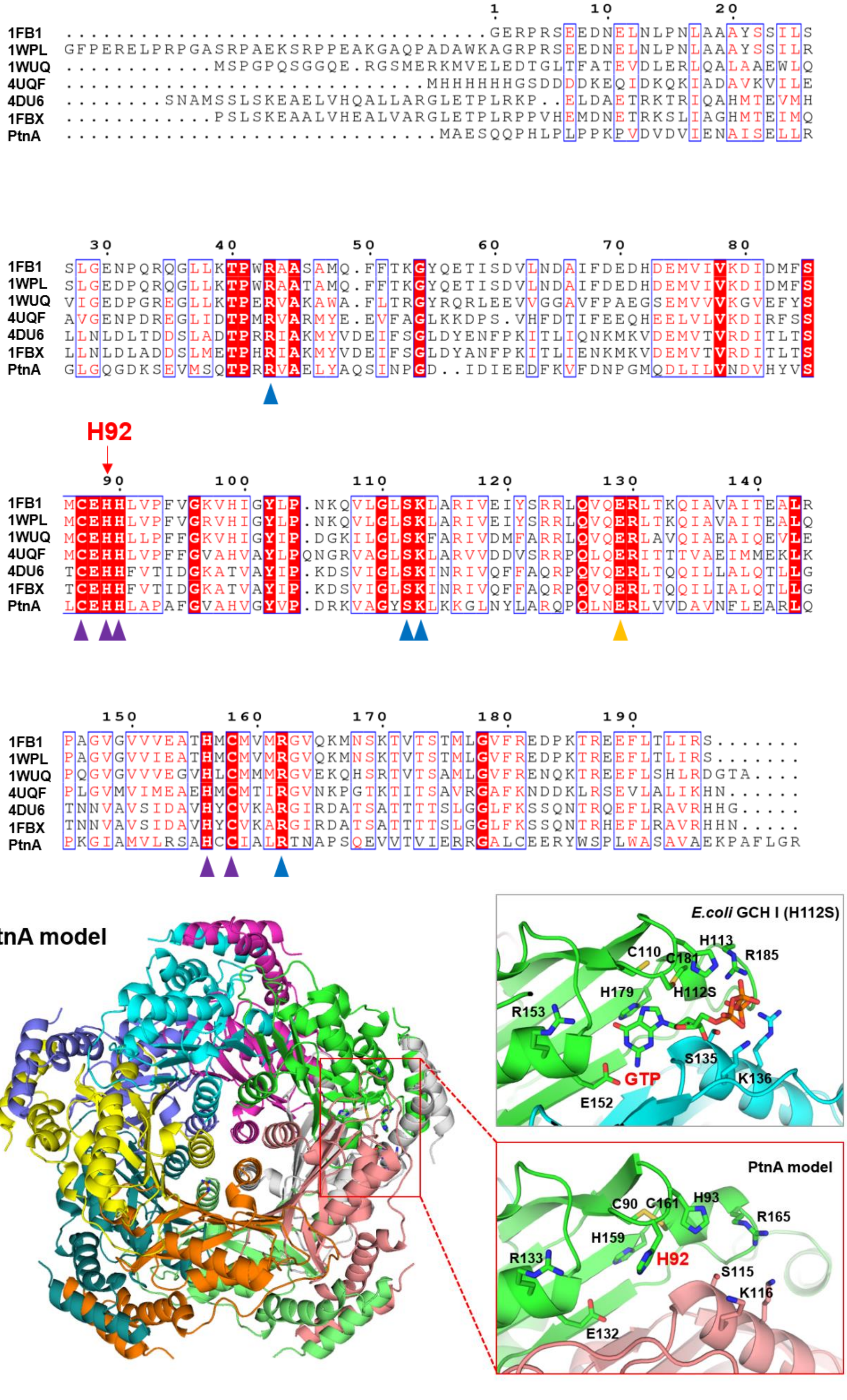

(**b**)

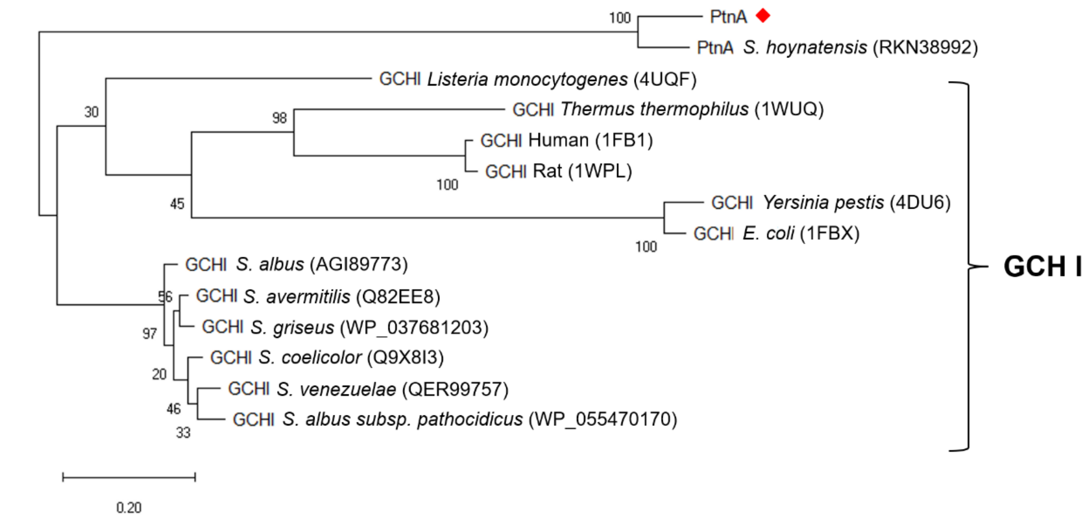

(**c**)

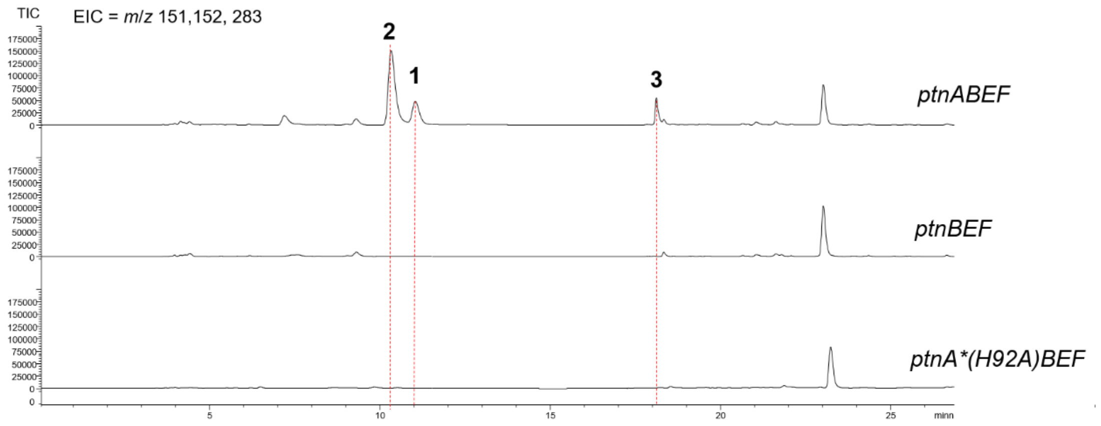

Supplementary Fig. 17. In silico and genetic study of PtnA. (**a**) Amino acid sequence alignment of PtnA with selected GCH I proteins. Conserved cysteine and histidine residues involving in zinc coordination and catalysis are indicated in purple-colored triangles. Residues that interacts with triphosphate group of GTP are indicated in blue color. Residue that was hydrogen-bonded to the amino group of guanine of GTP is indicated in orange color. GCH I used for sequence alignment are: 1FBX (*E. coli* GCH I), 1FB1 (human GCH I), 1WPL (rat GCH I), 1WUQ (*Thermus thermophilus* GCH I), 4UQF (*Listeria monocytogenes* GCH I), 4DU6 (*Yersinia pestis* GCH I). The PtnA homology model was generated by SWISS-MODEL[6] using 4UQF as a template. The H92 residue of PtnA that potentially involved in zinc-binding and catalysis is highlighted. (**b**) Phylogenetic analysis of PtnA. Note: The distribution of the *ptn* cluster appears to be very limited. PtnA forms a separate branch with the only close homolog (from *S. hoynatensis* strain KCTC 29097, accession number: RBAL01000015.1) we can identify in Genbank. This homolog is encoded by a putative operon similar to *ptnABCDE* (but lacking *ptnF*). (**c**) LC-MS analysis of engineered *S. albus* strains harboring different *ptn* genes revealed that the H92A variant of PtnA is inactive.

Supplementary Fig. 18.

(a)

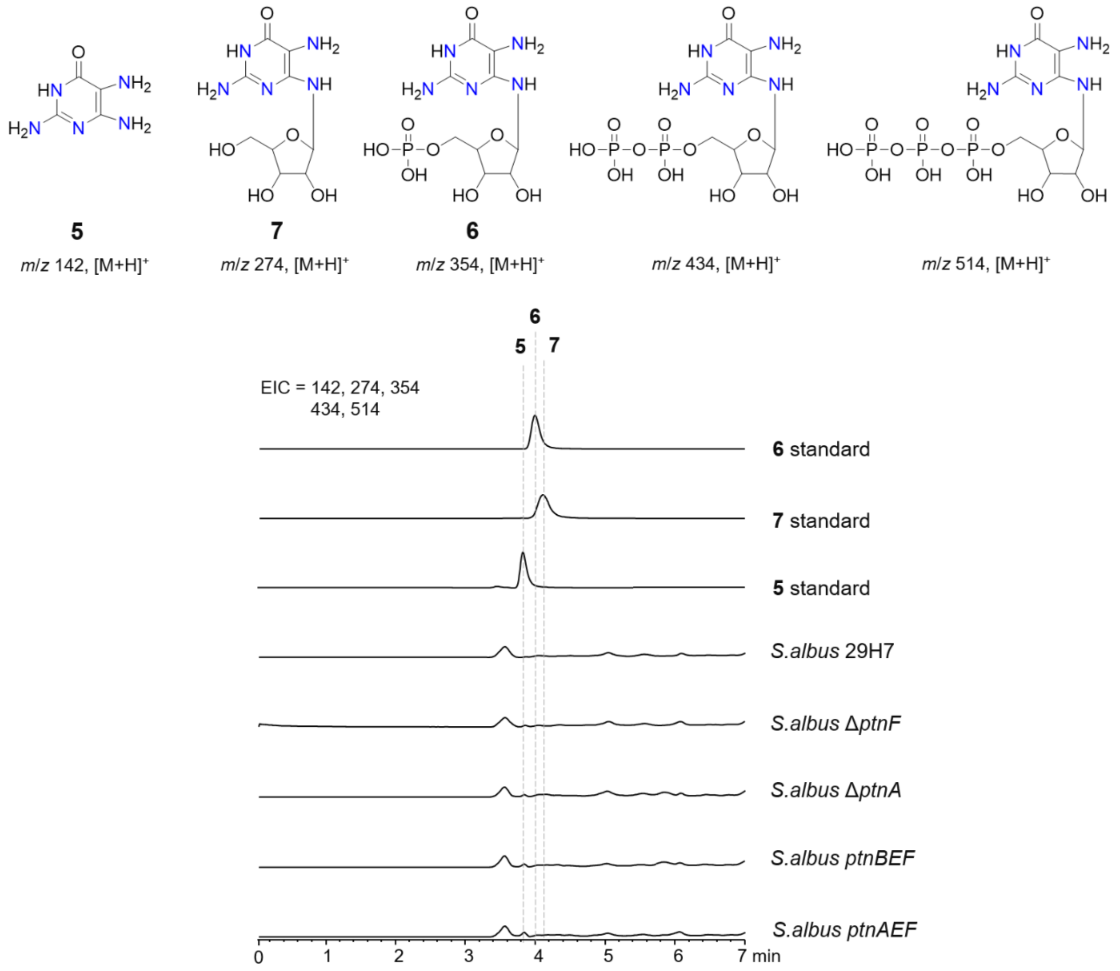

(**b**)

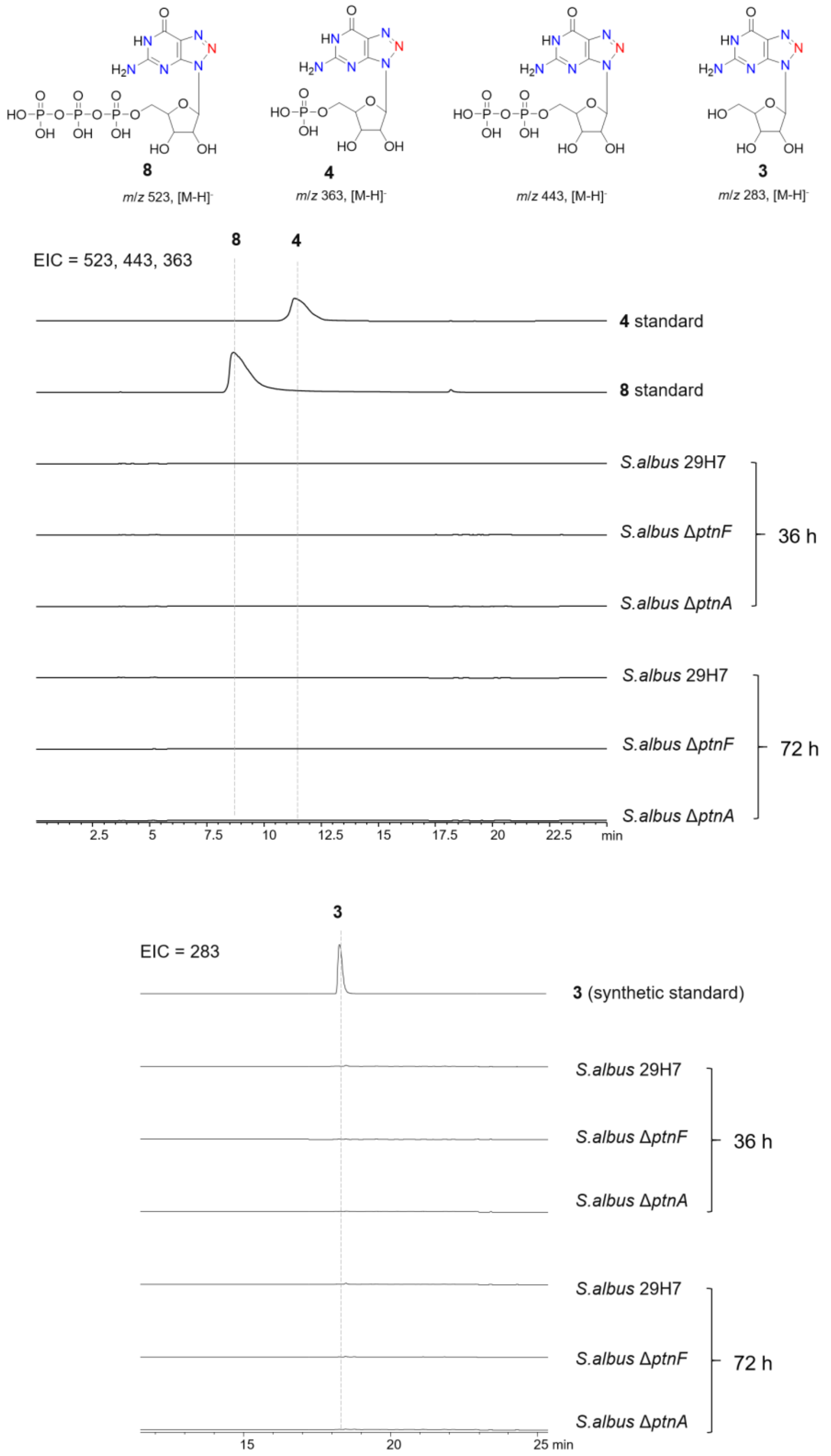

Supplementary Fig. 18. LC-MS analysis of culture supernatants (with or without DEA NONOate treatment) from selected *S. albus* strains for potential pathway intermediates. Authentic samples (~0.1 mM each) available were used as analytical standards. (**a**) LC-MS searching (positive mode) for ribosyl derivatives of **5** after 96 h's fermentation. (**b**) LC-MS searching (negative mode) for ribosyl derivatives of **1** after NONOate treatment of the culture supernatants from 36 h and 72 h's fermentation.

Supplementary Fig. 19.

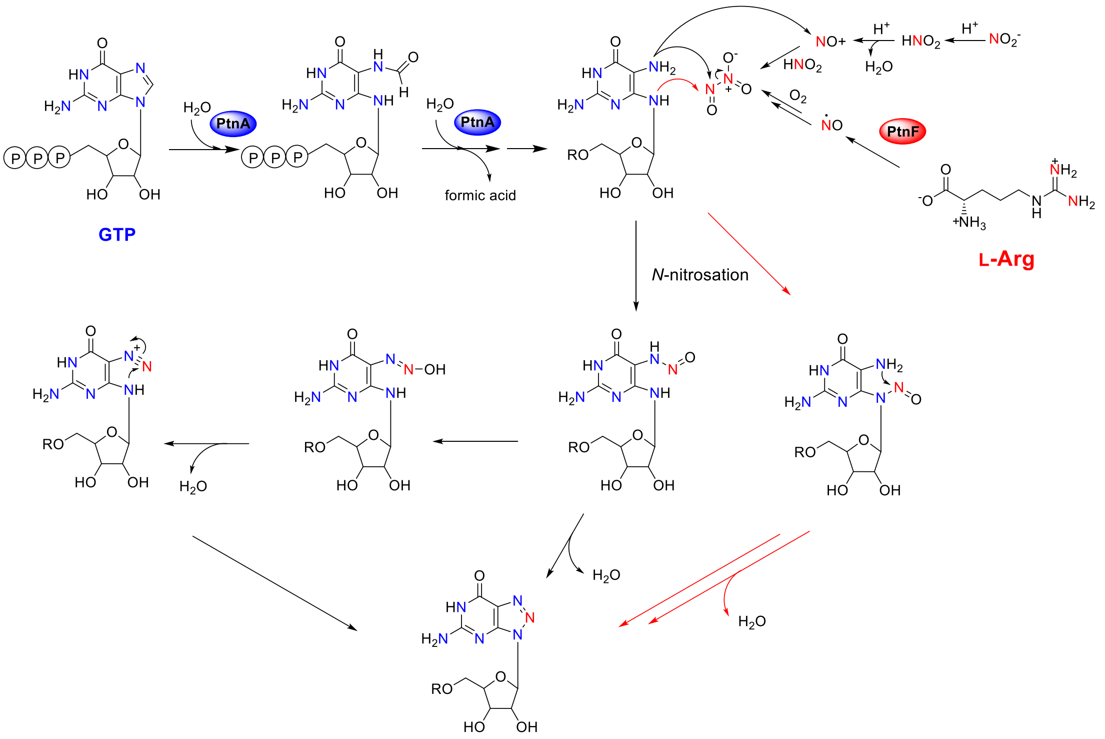

Supplementary Fig. 19. Proposed reaction mechanism for the enzymatic and nonenzymatic cascade leading to 1,2,3-triazole formation through *N*-nitrosative cyclization in the biosynthesis of 8-azaguanine. Note: it is possible that an enzyme process also mediates the triazole formation using nitric oxide directly *in vivo*.

Supplementary Fig. 20.

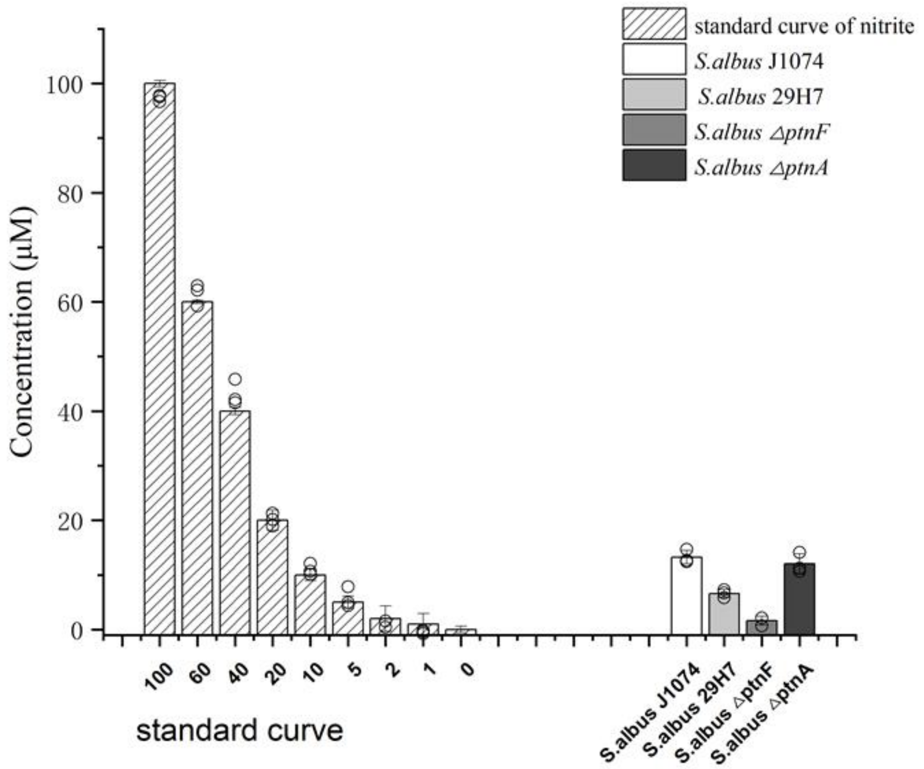

Supplementary Fig. 20. Determination of nitrite production in the culture supernatants (72 h) from selected *S. albus* strains including *S. albus* J1074, *S. albus* 29H7, *S. albus* Δ*ptnF* and *S. albus* Δ*ptnA*. Nitrite was quantified based on the Griess assay as described in Methods. Values are means of three independent experiments ± SD. Source data are provided as a Source Data file.

Supplementary Fig. 21.

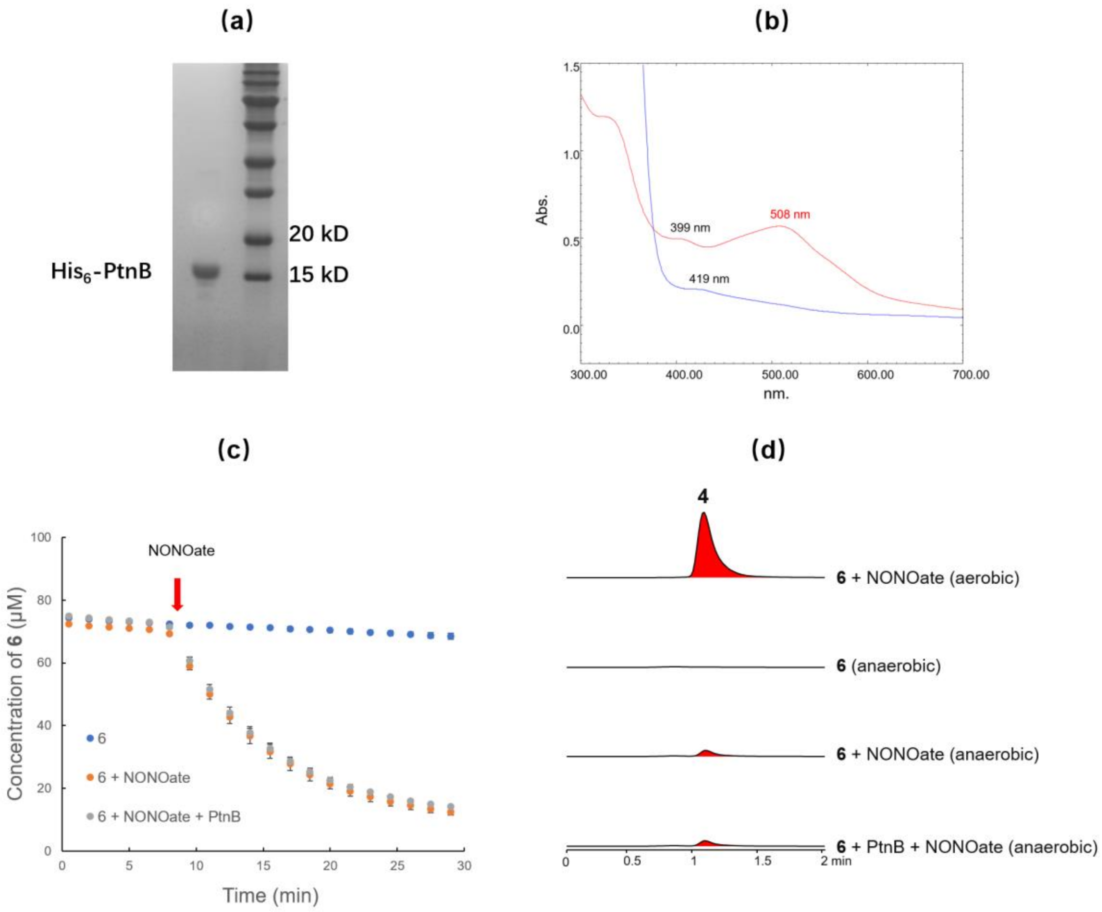

Supplementary Fig. 21. Analysis of the mixtures from *in vitro* incubation of PtnB and **6** in the presence of DEA NONOate. (**a**) SDS-PAGE of purified His-tagged PtnB. The experiment was repeated twice independently and the representative data was shown. (**b**) Spectroscopic analysis of as-isolated PtnB (450 µM, red line) and PtnB (450 µM) + sodium dithionite (10 mM) (blue line) revealing that PtnB is an iron-binding protein. The iron load of as-isolated PtnB is determined to be ~25% by the Ferrozine assay, which was performed as described previously[7]. Supplementation of ammonium iron (II) sulfate in the culture medium did not significantly increase the iron load. (**c**) Aerobic UV-vis spectroscopic analysis of **6** + PtnB (10 µM, as-isolated) + NONOate showed no triazole forming activity for PtnB. The red arrow indicates the time point when NONOate was added. The assays were performed similarly as described for **Fig.4c** in main text. The mixture of **6** + PtnB (10 µM) + NONOate displayed the same consumption rate of **6** as that of the control assay in which no PtnB was included. Values are means of three independent experiments ± SD. Source data are provided as a Source Data file. (**d**) UPLC-MS analysis of the anaerobically-prepared mixtures of **6** + PtnB (10 µM, reduced) + NONOate. The assays were performed similarly as described for **Fig.4d** in main text.

References:

1. Gräwert, T., Fischer, M. & Bacher, A. Structures and reaction mechanisms of GTP cyclohydrolases. *IUBMB Life* **65**, 310–322 (2013).
2. Madeira, F. *et al.* The EMBL-EBI search and sequence analysis tools APIs in 2019. *Nucleic Acids Res* **47**, W636–W641 (2019).
3. Robert, X. & Gouet, P. Deciphering key features in protein structures with the new ENDscript server. *Nucleic Acids Res* **42**, W320–W324 (2014).
4. WL DeLano. PyMOL: An Open-Source Molecular Graphics Tool - CCP4. (2002).
5. Frelin, O. *et al.* A directed-overflow and damage-control N-glycosidase in riboflavin biosynthesis. *Biochem J* **466**, 137–145 (2015).
6. Biasini, M. *et al.* SWISS-MODEL: modelling protein tertiary and quaternary structure using evolutionary information. *Nucleic Acids Res* **42**, W252–W258 (2014).
7. Guo, Y.-Y. *et al.* Molecular mechanism of azoxy bond formation for azoxymycins biosynthesis. *Nat Commun* **10**, 1–9 (2019).
